# Recent advances in predicting acute mountain sickness: from multidimensional cohort studies to cutting-edge model applications

**DOI:** 10.3389/fphys.2024.1397280

**Published:** 2024-06-24

**Authors:** Boyuan Wang, Shanji Chen, Jinfeng Song, Dan Huang, Gexin Xiao

**Affiliations:** ^1^ Beijing Xiaotangshan Hospital, Beijing, China; ^2^ Beijing Highland Conditioning Medical Center, Beijing, China; ^3^ The First Affiliated Hospital of Hunan University of Medicine, Huaihua, China; ^4^ Hunan Primary Digital Engineering Technology Research Center for Medical Prevention and Treatment, Huaihua, China; ^5^ National Institute of Hospital Administration (NIHA), Beijing, China; ^6^ Beijing University of Sport, Beijing, China

**Keywords:** high-altitude illnesses, acute mountain sickness, proactive health, comorbidity, prediction, cohort data, models, artificial intelligence

## Abstract

High-altitude illnesses, encompassing a spectrum of health threats including Acute Mountain Sickness (AMS), pose significant challenges to individuals exposed to high altitude environments, necessitating effective prophylaxis and immediate management. Given the variability in individual responses to these conditions, accurate prediction of high-altitude illnesses onset is of paramount importance. This review systematically consolidates recent advancements in research on predicting AMS by evaluating existing cohort data, predictive models, and methodologies, while also delving into the application of emerging technologies. Through a thorough analysis of scholarly literature, we discuss traditional prediction methods anchored in physiological parameters (e.g., heart rate, respiratory frequency, blood pressure) and biochemical markers, as well as the integration and utility of novel technologies such as biosensors, genetic testing, and artificial intelligence within high-altitude prediction research. While conventional pre-diction techniques have been extensively used, they are often constrained by limitations in accuracy, reliability, and multifactorial influences. The advent of these innovative technologies holds promise for more precise individual risk assessments and personalized preventive and therapeutic strategies across various forms of AMS. Future research endeavors must pivot decisively towards the meticulous identification and stringent validation of innovative predictive biomarkers and models. This strategic re-direction should catalyze intensified interdisciplinary cooperation to significantly deepen our mechanistic insights into the pathogenesis of AMS while refining existing prediction methodologies. These groundbreaking advancements harbor the potential to fundamentally transform preventive and therapeutic frameworks for high-altitude illnesses, ultimately securing augmented safety standards and wellbeing for individuals operating at elevated altitudes with far-reaching global implications.

## 1 Introduction

Plateau environments, defined as geographical regions with elevations surpassing 2,500 m, presenting extreme conditions of low oxygen, low pressure, low temperature, dry air, and strong ultraviolet radiation, which have a significant impact on the human cardiovascular system, respiratory system, metabolic system, and immune system. Especially due to hypoxia, the heart is one of the most metabolically active organs in the body, and myocardial metabolism is highly sensitive to ischemia and hypoxia. Prolonged and severe ischemia and hypoxia can directly lead to myocardial damage or even necrosis, endangering life (Maggiorini et al., 1990). Inhabiting, working, or traveling in such high-altitude settings increases the likelihood of individuals experiencing a range of pathophysiological responses related to altitude adaptation, with Acute Mountain Sickness (AMS) being one of the most prevalent. AMS arises from an inappropriate response to hypoxic conditions, characterized predominantly by persistent headaches and often accompanied by non-specific symptoms like nausea, vomiting, general fatigue, loss of appetite, and sleep disturbances (Hackett et al., 1976). Severe cases can progress to life-threatening High Altitude Pulmonary Edema (HAPE) and/or High Altitude Cerebral Edema (HACE).

Despite the abundance of theoretical knowledge and practical experience amassed in studying high-altitude illnesses, alongside the evolution of preventive measures and treatment strategies, accurately anticipating an individual’s predisposition to acute mountain sickness (AMS) remains a significant challenge. The application of artificial intelligence (AI), machine learning (ML), and deep learning (DL) techniques holds promise in scientifically forecasting AMS development upon entry into high-altitude environments. By particularly focusing on identifying and implementing targeted interventions for high-risk populations, these advanced methods have the potential to dramatically decrease the incidence and severity of AMS across the plateau population. However, to harness the full potential of AI, ML, and DL in this context, establishing a comprehensive and robust data repository is imperative as a foundational requirement.

This review aims to systematically and comprehensively survey the key research advancements made in recent years in the prediction of acute mountain sickness. By thoroughly examining and critically evaluating cohort study data, predictive models, biomarkers, and detection methods presented in existing literature, we also explore the potential applications of emerging technologies such as biosensing technology, genomic analysis, and artificial intelligence algorithms in AMS prediction. Our endeavor seeks to illuminate the cutting-edge dynamics of current AMS prediction methodologies, scrutinize unresolved issues and challenges, thereby providing authoritative and forward-looking reference points and practical guidance for global researchers and clinicians. Ultimately, this effort is expected to drive further advancement and development in the field of plateau medicine concerning the prevention and treatment of acute mountain sickness.

### 1.1 Overview of high altitude illnesses

High altitude illnesses, as a collective term for specific pathophysiological reactions occurring in high-altitude environments, encompass both acute and chronic forms (Basnyat, 2013). The acute manifestations mainly consist of Acute Mountain Sickness (AMS), High Altitude Pulmonary Edema (HAPE), and High Altitude Cerebral Edema (HACE) (Luks et al., 2017). AMS is an adaptive disorder triggered by rapid ascent to high altitude, characterized by symptoms such as headaches, nausea, vomiting, fatigue, insomnia, and loss of appetite (Luks et al., 2017). Key determinants of AMS onset include altitude, individual sensitivity, rate of ascent, duration of stay, physical exertion levels, and adaptation to hypoxic conditions (Schneider et al., 2002; Basnyat, 2013).

Webb et al. (2009) have highlighted that the human body partially adapts to high altitude through upregulated expression of HIF-1. Due to the thin air and low oxygen partial pressure at high elevations, humans often experience hypoxia. Given the inter-individual variability in tolerance to hypoxia, distinct pathophysiological responses emerge among individuals. Studies show that those who ascend above 2,500 m, particularly above 4,000 m, obese individuals, and those with a history of migraines are at significantly increased risk for AMS (Hirata et al., 1989; Lee et al., 2002; Simancas-Racines et al., 2018).

According to symptom severity, high altitude illnesses can be classified as mild, moderate, or severe (Ogilvie, 2001; Basnyat and Murdoch, 2003), as shown in Table 1. Mild cases present with minor headaches, insomnia, and decreased appetite; moderate patients exhibit these symptoms along with frequent vomiting, marked weakness, and palpitations, which can interfere with daily life but typically resolve within 2–3 days after onset. Severe high altitude illnesses manifest with intense vomiting, chest tightness, and drowsiness, severely compromising life safety and requiring urgent intervention; HAPE and HACE represent typical complications, involving pulmonary edema and cerebral edema respectively, which if left untreated, can lead to life-threatening consequences (Chen et al., 2023).

**TABLE 1:** Classification and symptoms of high altitude illnesses.

	Degree	Clinical manifestation
Acute Mountain Sickness (AMS)	Mild, self-limited	Headaches, nausea, vomiting, fatigue, insomnia, and loss of appetite
High Altitude Pulmonary Edema (HAPE)	Severe, life-threatening	Sports endurance decreased, labored dyspnea, dry cough, followed by resting dyspnea, coughing white foam sputum, and pink foam sputum in severe cases. Signs include mild fever, tachycardia, tachycardia, and wet rales
High Altitude Cerebral Edema (HACE)	Severe, life-threatening	Neurological symptoms: mental disorders, ataxia, unclear consciousness, hallucinations, and in severe cases, coma or even death (Li et al., 2018)

The Lake Louise Questionnaire was introduced in 1991 and was last modified in 2018. It is now the most commonly used scoring system used to assess AMS (Roach et al., 2018). It defines AMS as the presence of headaches in addition to three other symptoms, including gastrointestinal symptoms, fatigue/weakness, and dizziness/light headedness. Each symptom is appointed a point on a scale from 0 to 3, with 0 being no effect and 3 being severe. A total score of 3 or greater, with the presence of headaches, in a setting of rapid ascent to high altitude, is diagnosed as acute mountain sickness. The Lake Louise Questionnaire, although an effective assessment tool, has its limitation. It is a scoring system to standardize and diagnose AMS for use by investigators or research purposes, and it is not meant for clinical practice.

High altitude illnesses are characterized by: 1) rapid onset, with symptoms potentially emerging within 24–48 h upon reaching high altitude, as the body requires time to adapt to the hypoxic environment; 2) broad susceptibility, not restricted by factors like gender, age, or physical condition, as even healthy individuals may develop illness (Burtscher et al., 2021); 3) reversibility, with most symptoms resolving when altitude is reduced or appropriate treatment is given; and 4) significant individual variation, with substantial differences in the ability to adapt to high altitude environments among different individuals.

Understanding and appreciating these characteristics are crucial for timely, accurate, and targeted prevention and intervention of high altitude illnesses. To ensure the safety and health of individuals entering high altitude areas, effective control measures can include hypoxic preconditioning training, scientifically sound climbing plans, gradual ascending speeds, and pharmacological prophylaxis, among others (Chen et al., 2023; Toussaint et al., 2021).

Hypoxic preconditioning training involves exposure to low oxygen for a certain amount of time and intensity before hypoxic stress to generate endogenous protection and enhance the body’s tolerance to hypoxic hypoxia later on (Wang et al., 2021). Hypoxic pre-adaptation training can promote various adaptive changes in the body by simulating the conditions of high altitude and low oxygen, including increasing erythrocyte production, promoting blood vessel dilation and new blood vessel formation, optimizing metabolic mode, enhancing antioxidant and anti-inflammatory functions, adjusting gene expression, and helping the central nervous system adapt to low oxygen (Olfert et al., 2001; Baillieul et al., 2017; Viscor et al., 2018; González-Candia et al., 2022). Through the above mechanism, hypoxia pre-adaptation training can help the human body adapt to the low oxygen environment at high altitude in advance to a certain extent, reduce or avoid the occurrence of mountain diseases and related complications, and provide an effective prevention strategy for people entering high altitude areas. However, such training should be conducted under professional guidance to ensure safety and effectiveness (Girard et al., 2020).

### 1.2 Exploration of pathophysiological mechanisms in high altitude illnesses

**FIGURE 1:**
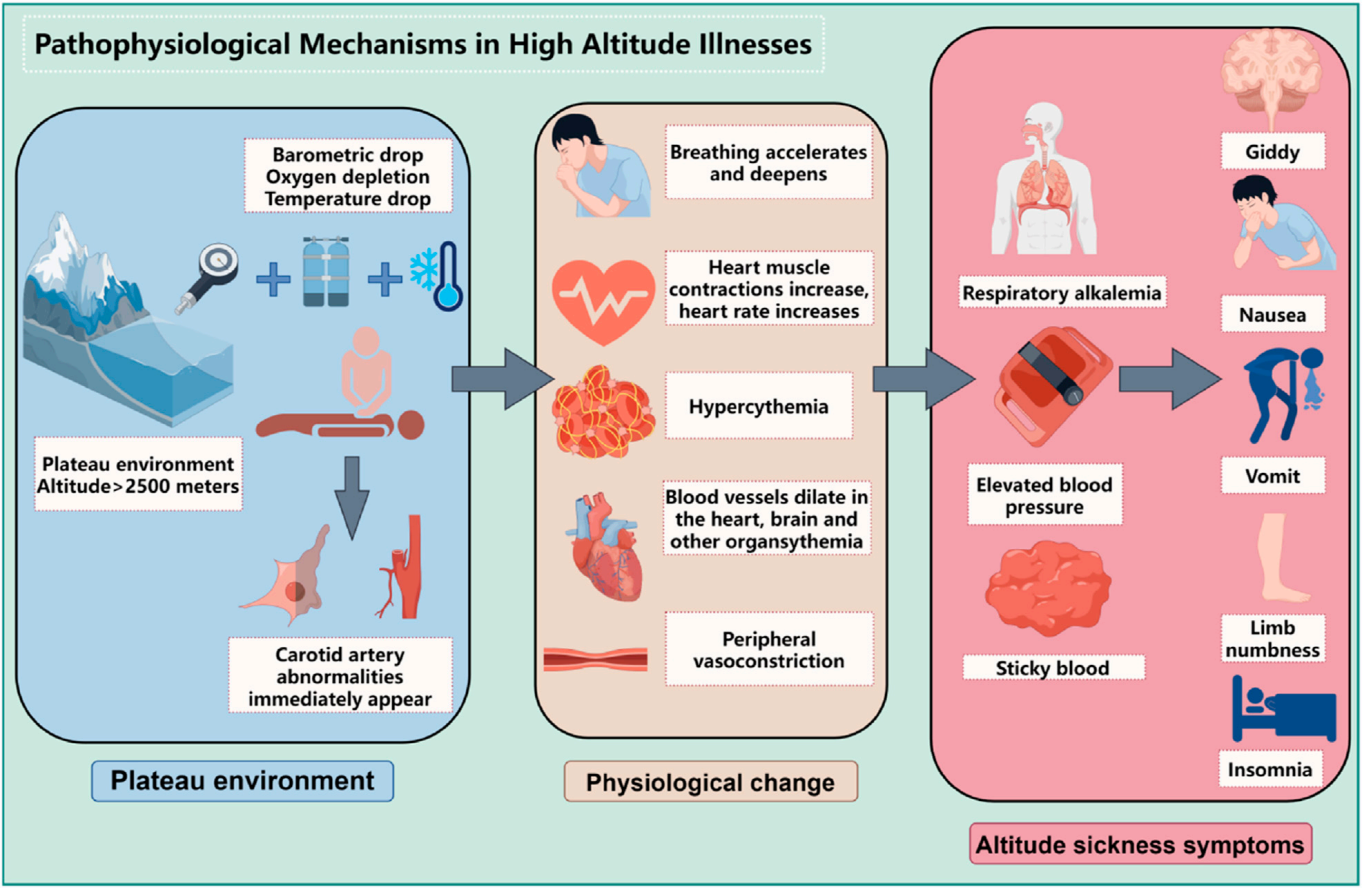
Pathophysiological mechanisms in high altitude illnesses (By figdraw).

The development and progression of high altitude illnesses are closely linked to the body’s adaptive responses to hypoxic environments at high altitudes, involving a series of complex pathophysiological changes, as shown in Figure 1. The following will elaborate on these critical processes:

#### 1.2.1 Oxygen supply limitation

**FIGURE 2:**
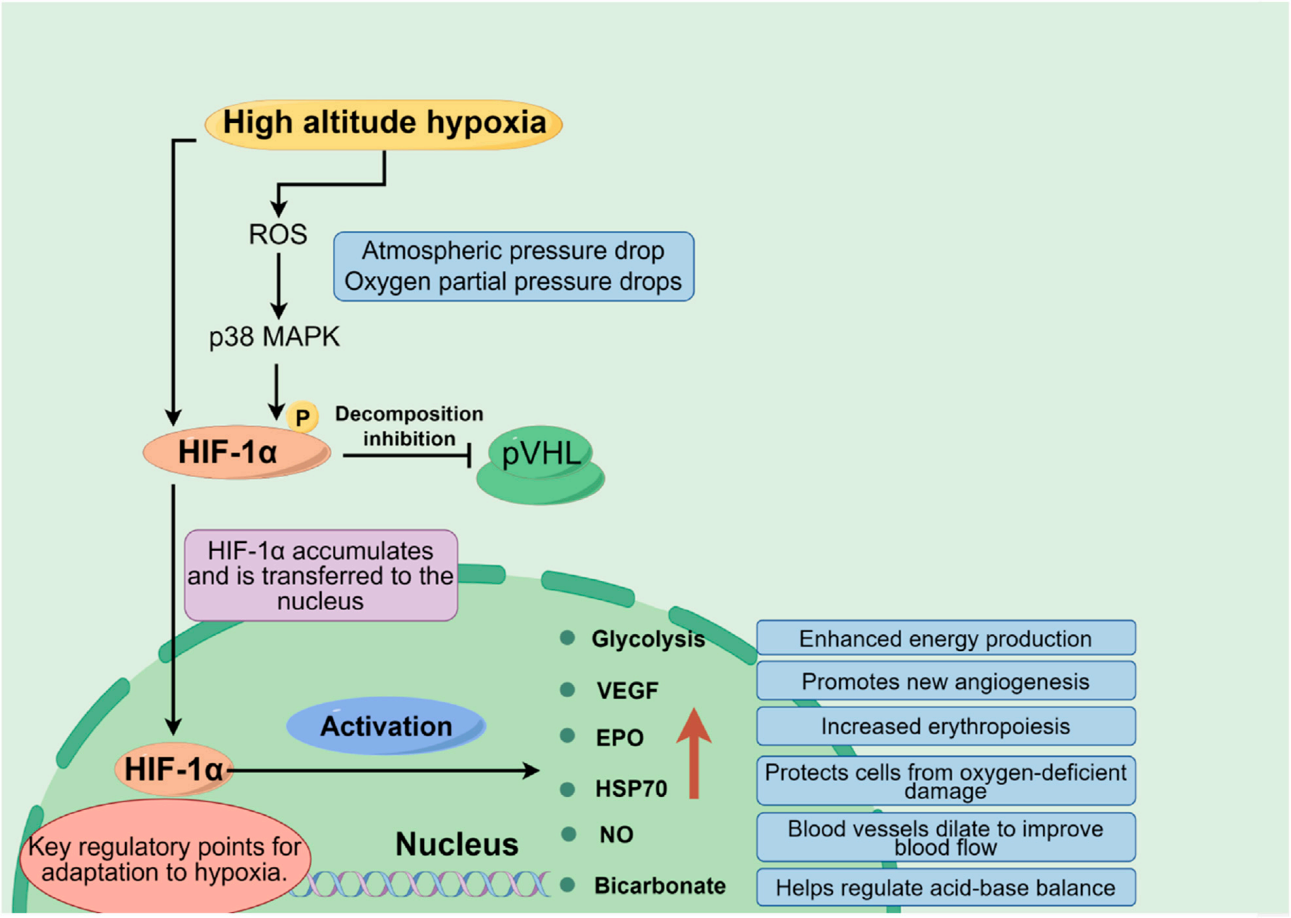
Oxygen supply and demand balance mechanism in plateau environment (By figdraw).

At high altitudes, with decreasing atmospheric pressure, the partial pressure of oxygen reduces correspondingly, which lessens the oxygen diffusion gradient across the alveolar-capillary interface, leading to a significant decrease in the amount of oxygen carried by the blood (lowering oxygen saturation). Insufficient oxygen supply constitutes the core pathophysiological basis for high altitude illnesses. Hypoxia-inducible factors (HIFs), particularly the HIF-1α subunit, play a pivotal role here. Under low oxygen conditions, HIF-1α stabilizes and activates, regulating the expression of thousands of genes including glycolytic enzymes, Vascular Endothelial Growth Factor (VEGF), and Erythropoietin (EPO) (Dzhalilova and Makarova, 2020). Additionally, molecules like Heat Shock Protein 70 (HSP70) and Nitric Oxide (NO) also participate in modulating an individual’s tolerance to hypoxia, as shown in Figure 2.

#### 1.2.2 Compensatory physiological responses to hypoxia

In response to the hypoxic challenge at high altitude, the body initiates several adaptive physiological responses:

Respiratory System: By increasing respiratory rate and depth to enhance oxygen uptake and carbon dioxide elimination.

Cardiovascular System: Within days of initial exposure to hypoxia, autonomic nervous system adjustments result in increased heart rate and cardiac output, optimizing oxygen delivery to tissues throughout the body, thus protecting the heart from excessive workload due to increased metabolic demands.

Blood System: Under the stimulation of chronic hypoxia, the expression of hypoxia inducible factors is activated, thereby stimulating the kidneys and liver to secrete a large amount of EPO (Du and Bai, 2015). EPO activates JAK2 and STAT-5 related signaling pathways by binding to EPO receptors in hematopoietic organs, further upregulating the expression of membrane proteins and hemoglobin, thereby inducing massive proliferation of red blood cells and increasing the number of red blood cells in the bloodstream. This can improve tissue oxygen supply to a limited extent, making the body less prone to pathological changes. As hypoxia worsens, EPO regulation becomes dysregulated, stimulating the proliferation of immature red blood cells at different stages of bone marrow development, and changing the size and shape of red blood cells to meet physiological needs, expanding the contact surface with oxygen, effectively improving tissue hypoxia. But when severe hypoxia occurs, the number of red blood cells increases, and the compensatory significance of the above changes into harmful effects, causing an increase in blood viscosity, a decrease in cell deformability, and difficulty for cells to pass through microvessels, further leading to tissue hypoxia, a vicious cycle, and ultimately the occurrence of HAPC.

Renal System: During acute hypoxia, activation of the adrenergic system leads to tachycardia; meanwhile, hypoxia-induced pulmonary vasoconstriction raises pulmonary artery pressure, and the kidneys respond by secreting erythropoietin to stimulate erythropoiesis in bone marrow, further augmenting red blood cell numbers (Richalet et al., 2023).

#### 1.2.3 Increased blood viscosity and hemodynamic changes

Due to the increased number of red blood cells and heightened hemoglobin concentrations, blood viscosity significantly rises under high-altitude conditions, presenting as increased resistance to blood flow, exacerbating the heart’s workload, and potentially affecting microcirculation and overall circulatory efficiency (Semenza, 1994; Savioli et al., 2022).

#### 1.2.4 Tissue damage and pathological processes

Long-term or severe hypoxia can trigger various tissue damages and pathological processes:

Cellular Oxidative Stress: Cellular oxidative stress is a pathophysiological state in which the balance between intracellular oxidation and antioxidant action is upset in favor of the oxidative process, resulting in the abnormal accumulation of reactive oxygen species (ROS) such as superoxide anions (O2 ⁻), hydrogen peroxide (H₂O₂), hydroxyl radicals (OH⁻), etc. This process not only affects normal cell metabolism, but also directly or indirectly damages biological macromolecules, including proteins, lipids, nucleic acids, etc., thus interfering with many aspects of cell signaling, energy production, gene expression, and cell cycle regulation (Tan et al., 2018). Under low oxygen conditions, the oxygen supply to cells is limited, and the function of the electron transport chain of the mitochondria may be impaired, leading to electron leakage and promoting ROS production. At the same time, low oxygen can also affect the antioxidant defense system, such as reducing the activity of enzymes such as superoxide dismutase (SOD), catalase (CAT) and glutathione peroxidase (GPx), making it more difficult for cells to clear ROS, thus exacerbating oxidative stress (Zaarour et al., 2023). Oxidative stress is not only closely related to the occurrence and development of a variety of diseases, such as cardiovascular diseases, neurodegenerative diseases, diabetes, cancer, etc., but also participates in the aging process (Arfin et al., 2021). In recent years, intervention strategies for oxidative stress have become a research hotspot. These include the use of antioxidants, regulation of the balance between intracellular ROS production and clearance, and drug development targeting specific ROS-related signaling pathways (Leyane et al., 2022). For example, certain natural compounds such as flavonoids (such as genistein) have been found to reduce the damage of cells caused by oxidative stress, which may be related to their direct removal of free radicals or regulation of the antioxidant defense system (Alorda-Clara et al., 2022).

Inflammatory Response: The inflammatory response is a complex biological defense mechanism designed to eliminate harmful irritants and promote tissue repair. The latest scientific research continues to deepen our understanding of this process. Hypoxia, a lack of oxygen supply, is one of the key factors that triggers the inflammatory response. Under hypoxia, cells induce the release of pro-inflammatory mediators through a variety of pathways, including but not limited to tumor necrosis factor-α (TNF-α), interleukin-1β (IL-1β), interleukin-6 (IL-6), and vasoactive mediators such as histamine and bradykinin (Malkov et al., 2021).The release of proinflammatory mediators initiates a cascade of reactions that includes the following key steps (Gusev E et al., 2021; Soares et al., 2023): vascular response; Leukocyte mobilization; Cytokine storm; Tissue repair and remodeling. Recent studies have also focused on the fine regulatory mechanisms of inflammatory response, such as the activation of NLRP3 inflammasome in sensing various danger signals (Seoane et al., 2020), and the complex role of Hypoxia-inducible factors (HIFs) in regulating inflammation and immune response (Foglia et al., 2021). Hypoxia induces the release of pro-inflammatory mediators, triggering an inflammatory cascade that results in vasodilation, increased vascular permeability, and other inflammatory manifestations.

Tissue Edema: Examples include high altitude cerebral edema (HACE) and high altitude pulmonary edema (HAPE), which are severe complications of high altitude illnesses. These occur mainly due to circulatory disturbances, increased capillary leakage, and fluid accumulation (Scoggin et al., 1977; Dehnert et al., 2005).

#### 1.2.5 Complications and their risk

Severe cases of high altitude illness can lead to potentially fatal complications:

High Altitude Pulmonary Edema (HAPE): HAPE is non cardiogenic pulmonary edema caused by acute severe hypoxia, which leads to pulmonary vein vasoconstriction, causing a sharp increase in pulmonary arterial pressure, local hyperperfusion, increased pulmonary blood flow, and imbalanced ventilation blood flow ratio. In addition, fluid leaks into the pulmonary interstitium and alveoli through pulmonary capillaries, hindering normal gas exchange (Choudhary et al., 2022).

High Altitude Cerebral Edema (HACE): HACE is considered to be the final stage of acute high-altitude reaction. In addition to worsening symptoms such as headache, dizziness, and vomiting mentioned above, ataxia, mental confusion, varying degrees of consciousness disorders, fever, and in severe cases, brain herniation can occur, endangering life (Li et al., 2023).The exact pathophysiological mechanisms underlying the development of AMS and HACE have not been fully determined. One of the main theories explaining the development of AMS is the increase in intracranial pressure. There are two important factors that cause an increase in intracranial pressure: the generation of cerebral edema and an increase in intracranial blood flow. Vascular edema (accumulation of extracellular water due to increased blood-brain barrier permeability) and cytotoxic edema (disruption of Na+/K + pump function on the cell membrane) are considered the underlying mechanisms of HACE (Bailey et al., 2009).

The pathophysiology of high altitude illnesses encompasses limited oxygen supply, hypoxia-induced physiological adaptations and compensatory responses, changes in blood rheology, and the development of related tissue damage and complications. A deep understanding of these mechanisms is essential for guiding the formulation of prevention strategies, early diagnosis, and effective treatment of high-altitude illnesses, both theoretically and practically.

## 2 Current status and advances in cohort studies of high altitude illness populations

Cohort studies, due to their exceptional ability to unravel complex interplays between compounded exposures such as high-altitude environments, lifestyle habits, and genetic components with health outcomes, have been universally recognized as a preeminent research approach in exploring etiological mechanisms and formulating strategies for disease prevention and management (Li and Lyu, 2015). Within the realm of high altitude illness research, the creation and meticulous examination of long-term, prospective cohorts among populations chronically exposed to high altitudes allow scientists to meticulously discern the fundamental patterns of disease manifestation and progression, along with related risk factors. The data harvested from these studies act as a pivotal resource and groundwork for developing predictive models. Researchers utilize long-term, forward-looking cohort methodologies to methodically gather and interpret data concerning individuals dwelling in high-altitude settings, with the aim to uncover the incidence patterns, developmental trends, and risk factors associated with high altitude illnesses, as well as to assess the practicality and effectiveness of prophylactic and treatment strategies.

(1) Study Subjects and Scope: Currently, cohort studies on high altitude illness populations encompass a diverse range of groups, including but not limited to permanent residents living in high-altitude regions like the Tibetan Plateau, workers frequently traveling between low and high altitudes, professional and amateur athletes participating in mountaineering expeditions, and tourists visiting high-altitude destinations. The diversity of study subjects helps to comprehensively understand the differences in adaptability and susceptibility among various populations to high-altitude environments.
(2) Incidence and Severity Research: Several cohort studies show that the incidence of high altitude illnesses (such as Acute Mountain Sickness, Chronic Mountain Sickness, High Altitude Pulmonary Edema, and High Altitude Cerebral Edema) increases with rising altitude and exhibits significant individual variability (Hackett et al., 1976; Gongga et al., 2014; Wang et al., 2014). Through longitudinal follow-ups of large samples, research teams have quantified changes in the frequency and severity of high altitude illnesses at different altitudes, providing key data for risk prediction models.
(3) Exploration of Influencing Factors: Researchers have thoroughly investigated and statistically analyzed a variety of potential factors affecting the occurrence and progression of high altitude illnesses. These factors include modifiable habits such as lifestyle, physical fitness, and nutritional status, as well as non-modifiable biological traits like genetic background, age, and gender. For instance, certain gene polymorphisms have been associated with an individual’s capacity to adapt to hypoxic high-altitude environments, thereby influencing their risk for high altitude illnesses (Zhao et al., 2023).

The activation of RAAS at high altitudes, where hypobaric hypoxia leads to reduced oxygen availability, plays a pivotal role. Under such conditions, there is an increase in renin secretion from the kidneys, initiating a cascade that results in the production of angiotensin II (Ang II) and subsequent aldosterone release. Ang II, a potent vasoconstrictor, contributes to the maintenance of blood pressure but can also lead to pulmonary vasoconstriction and increased vascular permeability, predisposing individuals to HAPE. Meanwhile, aldosterone enhances sodium retention and fluid accumulation, potentially exacerbating the edematous processes observed in both HAPE and HACE (Ferrario and Mulick, 2017; Hundemer and Sood, 2021). Furthermore, recent studies have highlighted the potential therapeutic benefits of RAAS inhibitors in mitigating the severity of altitude sickness symptoms, emphasizing the system’s critical involvement in the disease progression (Duo et al., 2023; Richalet et al., 2023).

(4) Evaluation of Prevention and Intervention Measures: Based on cohort data, scientists assess various methods for preventing and treating high altitude illnesses, such as drug prophylaxis (e.g., acetazolamide and dexamethasone use), stepwise acclimatization training, physiological and psychological conditioning, and rational dietary supplementation strategies. The research findings provide a scientific basis for developing targeted and effective health protection plans for high-altitude settings.

Cohort studies on high altitude illness populations play a vital role in etiological and epidemiological research, continually enriching and deepening our understanding of physiological and pathological changes in humans under high-altitude conditions. They significantly propel the development of high-altitude medicine and are of great importance for ensuring the health of people living in high-altitude regions and improving work efficiency in these areas. Currently, both China and other parts of the world have established numerous population cohorts to further this research agenda.

### 2.1 Status of cohort studies in China

In China, cohort studies have made significant progress in different geographical regions across the country, as shown in Table 2. For instance, the “Southwest Region Natural Population Cohort Study” project, led by Professor Xiaosong Li from the West China School of Public Health/West China Hospital Fourth Affiliated Hospital, has made outstanding contributions to establishing a natural population cohort with unique environmental features, ethnic diversity, and disease distribution characteristics in the Southwest region. Since its inception in 2017, the “Southwest Cohort” has successfully completed baseline surveys and a follow-up visit with an impressive 93% retention rate, collecting biological samples from 112,000 individuals. It has also established baseline databases, follow-up databases, and a biobank covering the natural populations across five provinces and cities in the Southwest region, marking a major breakthrough in large-scale natural population cohort research in multi-ethnic areas of Southwest China. Additionally, this study innovatively developed a high-standard technical specification system for cohorts applicable to China’s multi-ethnic regions. Through biomedical big data mining and analysis, it revealed the prevalence patterns and risk factors of prominent health issues in the Southwest region, translating scientific findings into policy, thereby providing a practical and feasible “Southwest Solution” for precision prevention and proactive health strategies, garnering international recognition for its research outcomes (Zhao et al., 2023).

**TABLE 2:** Status of cohort studies in China.

Team	Time	Participant	Altitude	Conclusion
Professor Li Xiao song’s team from West China University of Public Health/Fourth Affiliated Hospital of West China Hospital Zhao et al. (2023)	2017	A Study on the Natural Population Queue in the Southwest Region	Five provinces and cities in the southwest region	We conducted biomedical big data mining and analysis from multiple exposure dimensions, elucidated the prevalence patterns and risk factors of prominent health problems in Southwest China, and contributed to the precise prevention and proactive health plan in Southwest China, achieving a high level of research results
The Northwest Natural Population Queue Research Group of the Medical Department of Xi’an Jiaotong University Dang et al. (2023)	2017–2019	117,644 natural populations of multiple ethnic groups (Han, Hui, Uyghur, Kazakh, and Tibetan) aged 35–74	Establishing natural population queues in the northwest region in Shaanxi Province, Gansu Province, Qinghai Province, Ningxia Hui Autonomous Region, and Xinjiang Uygur Autonomous Region	This provides a research platform for in-depth research on the relationship between environmental, lifestyle, and genetic factors and important high-risk chronic diseases. It also provides important epidemiological evidence for clarifying the causes and long-term health hazards of chronic diseases among different ethnic groups in the region, and provides a reference for the development of chronic disease prevention and control strategies in the northwest region
Professor Han Shurong’s team from Shandong University Han et al. (2020)	2006–2018	286 Qinghai Tibet Railway maintenance personnel	Altitude>2,500 m	More than 60% of participants experience cardiac abnormalities after working in high-altitude areas, mainly due to right heart enlargement and left ventricular diastolic dysfunction. Employment age and work height are important risk factors for heart abnormalities
The research team led by Dr. Chen Renzheng from the Second Affiliated Hospital of the Third Military Medical University (now Army Medical University) of the Chinese People’s Liberation Army Cardiovascular Disease Research Institute Chen et al. (2021)	2019	72 healthy subjects aged 20–30	Altitude: 4,100 m	AMS patients have lower pulse pressure (PP) arterial elasticity (Ea) at low altitudes. These baseline indicators of LA vasodilation are closely related to AMS, which may explain the higher severity of headache in subjects with higher LA PP.
Professor Zhang Laiping’s research group at Xinqiao Hospital of Army Medical University Zhang et al. (2020), Chen et al. (2021), Dang et al. (2023)	2012	83 healthy young male participants from China	Lhasa (altitude 3,700 m), exposed to high altitude for 24 h	LVEDD is an independent risk factor for AMS, with a higher baseline LVEDD in the plain, which may indicate an increased incidence of AMS. After exposure to high altitude, circulatory hemodynamics and psychological states such as anxiety and fatigue may be involved in the occurrence of AMS.
Research Team of the Sixth Traditional Chinese Medicine Center of the 301 General Hospital of the People’s Liberation Army of China Li et al. (2023)	2022	80 male officers and soldiers aged 18–35 from a certain unit participating in high-altitude training teams	Rapidly entering the plateau (above 3,000 m above sea level)	Danqi Jing Granules can reduce the score of traditional Chinese medicine syndrome grading and quantification table, and improve the clinical symptoms of people who rush to high altitude; Perhaps by reducing serum TNF- α. The level of IL-6 plays a protective role in the occurrence of AMS in individuals exposed to acute high-altitude conditions
The team led by Tang Zhiai from Xinjiang Military Region General Hospital Tang et al. (2020)	2019	Health examination data of 1,350 long-term military personnel stationed in high-altitude areas	Altitude (1,000–2,900 m)	Soldiers who have been exposed to high-altitude environments for a long time have shown compensatory changes in their various systems, especially in the cardiovascular and respiratory systems. The abnormal rates of hemoglobin elevation, sinus bradycardia, and high diastolic blood pressure are as high as 56.07%, 40.96%, and 6.59%, respectively. These abnormal manifestations are significantly correlated with the duration of high-altitude residency and military altitude
BaiXueyazi research team from the Medical School of Tibet University Baixue et al. (2023)	2022–2023	106 young males from Area A (Group A) and 145 young males from Area B (Group B) immigrated to the plateau at similar altitudes on the Qinghai Tibet Plateau	4,270 m in Zone A and 4,295 m in Zone B	In addition to altitude, the microclimate environment in high-altitude areas also plays a crucial role in influencing the prevalence of high-altitude diseases among mobile populations
Pasang Zhuoma research team from the Medical School of Tibet University Gongga et al. (2014)	2014	865 Tibetan youth aged 15–25 at different altitudes (422 males and 443 females)	Altitude (2,700, 3,650, 4700 m)	The blood pressure of Tibetan youth living in Xizang is generally low, suggesting that Tibetan altitude adaptation does not increase the oxygen uptake capacity of the body by increasing blood pressure, but this result needs further research and verification to explore whether Tibetan people at different altitudes have different altitude hypoxia adaptation modes and adaptation mechanisms
Pasang Zhuoma research team from the Medical School of Tibet University Tsring et al. (2017)	2017	1,200 cadres and employees (579 Tibetan residents and 536 immigrants)	Altitude 3,658 m	The natives in Xizang have better adaptability to high altitude than the migrants, but there are still some injuries to various systems of the body caused by the inadequate adaptation to high altitude
Li Juan Research Team of Plateau Medical Research Center of Xizang University Li et al. (2019)	2019	673 healthy Tibetan residents living in different altitude gradients	The elevations are 2,227, 3,660, 4,500, and 5,018 m, respectively	As altitude increases, lung ventilation function compensates for an increase to meet the body’s oxygen needs. However, when the altitude reaches a certain level, lung ventilation function no longer increases accordingly. It can be inferred that there is a limit to the compensatory function of lung ventilation, indicating that when the altitude exceeds the human body’s regulatory capacity, lung function will no longer be enhanced
Ciren Zhuoma and Penba's Research Team of Plateau Medical Research Center of Xizang University Ciren et al. (2021)	2021	872 Tibetan residents living in different altitude gradients	Altitude: (2,516, 4,500, 3,660, 5,018) meters	The detection rates of overweight and obesity first increase and then decrease with the increase of altitude, and the BMI shows a significant downward trend above 4,500 m altitude
The High Altitude Military Medical College of the Third Military Medical University, led by Gao Yuqi‘s team Yang et al. (2011)	2008–2010	73 non Qinghai health examination personnel aged 20–50 from three units stationed in Xining	Altitude 2,200 m	Individuals residing in the sub high altitude area of 2,200 m have elevated levels of triglyceridemia in 35.6%, hyperuricemia in 32.9%, and alanine aminotransferase in 10.9%, resulting in mild liver function damage
The High Altitude Military Medical College of the Third Military Medical University, led by Zhou Qiquan’s team Wang et al. (2014)	2014	Blood biochemical indicators of 693 male cadres undergoing physical examination	Altitude 2,800 m, 3,400 m, 4,500 m	It was found that as altitude increases, the incidence of hyperuricemia also increases, reaching 28.88%, 50.37%, and 69.86%, respectively. And the incidence of hyperuricemia in patients with high altitude polycythemia, elevated blood creatinine, and overweight is (1.69–2.21)
Researchers at the High Altitude Military Medical College of the Third Military Medical University, led by Yi Yuan Yue Yi et al. (2017)	2017	42 healthy young male officers and soldiers aged 20–48	43 h by train from the plain (500 m above sea level) to the plateau (3,658 m above sea level)	During acute high-altitude exposure, the right ventricular function of young male soldiers is impaired, and the left ventricular function is compensated. Observing PASP and CO using echocardiography may help screen susceptible populations of AMS in plain areas。
The High Altitude Military Medical College of the Third Military Medical University, led by Gao Yuchi‘s team Zhou et al. (2017)	2017	956 male soldiers continuously stationed on the plateau and 587 male soldiers stationed on the plains were selected from a certain military region	Plain altitude 150–1,700 m, plateau altitude 3,200–5,400 m	The high-altitude environment seriously affects the sleep quality of high-altitude troops and has a strong correlation with CMS. While improving sleep quality, it is necessary to strengthen the prevention and treatment of CMS.
He Feng Research Team of the Medical Department of the General Hospital of the Xizang Military Region He et al. (2013)	2009	487 male subjects aged 16–48	Residing at an altitude of 4,500 m	High altitude low-pressure hypoxia has a serious impact on the sleep quality of people who migrate to plains at altitudes above 4,500 m. The continuity of their sleep is disrupted, and their sleep structure is disrupted. Moreover, the higher the altitude and the longer the time they migrate to the plateau, the poorer the sleep quality
Yuan Zhimin and others from Ali District People’s Hospital in Xizang Yuan et al. (2023)	2019–2021	207 individuals (114 Tibetans and 93 Han Chinese) aged 18–60 with a continuous 5-year history of high-altitude residency underwent physical examination	Altitude 4,300 m	In high-altitude areas, Han immigrants experience long-term pressure changes compared to Tibetans. The main differences are reflected in the blood system, liver and kidney function, etc., providing basic data for further research on the health status of high-altitude populations
Fudan University’s School of Life Sciences, the research team led by Wang Jiucun Wang et al. (2018)	2018	318 healthy Han Chinese males aged 18–35 in the plain	Within 72 h, from sea level (50 m above sea level) to altitude of 3,700 m	The high incidence rate of HAH was 74.84%. The subgroup analysis of HAH and non HAH verified that blood oxygen saturation and heart rate were independent risk factors for HAH, and blood urea nitrogen was first confirmed as independent risk factors for HAH.
Liu Zhengkui Team, Institute of Psychology, Chinese Academy of Sciences Wang et al. (2019)	2016	24,141 adult residents from Yushu Tibetan Autonomous Prefecture	Altitude 4,493 m	The prevalence of depression in high altitude areas is as high as 28.6%, and the incidence rate of depression is positively correlated with altitude. In addition, gender, age, income level, education level, and self-awareness have also been identified as risk factors for depression
Wang Yaxuan’s team from the Department of Psychiatry at the Medical School of the National Defense Medical Center Wang et al. (2023)	2000–2015	127 AMS patients and 1,270 healthy controls	——	The AMS group is associated with anxiety, depression, bipolar disorder, sleep disorders, post-traumatic stress disorder/acute stress disorder, mental disorders, and substance related disorders (SRD). In a 16 years long-term follow-up study, there was an association between AMS and an increased risk of mental illness
Ma Liguang from the National Family Planning Research Institute and Chen Qiuhong from the Central Laboratory of Qinghai Provincial Cardiovascular Disease Vocational Hospital, Ma et al. (2018)	2006–2008	Epidemiological data of 288,066 children (aged 4–18)	Altitude 3,000–5,000 m	The incidence of coronary heart disease shows a significant spatial clustering pattern in QTP. The clustering pattern of coronary heart disease subtype prevalence has statistical regularity and can provide convenient clues for environmental risk factors. The results can provide a basis for formulating coronary heart disease prevention strategies and reducing the incidence rate of coronary heart disease in high altitude areas of China
Researchers from the China Institute of Sport Science and the Tibet Institute of Sports Science, including Fan Chaoqun and Nie Mingjian among others Fan et al. (2021)	2019	2,000 students aged 9–18	At an altitude of 3,500 m, different grades in a certain college	Teenagers living above an altitude of 3,000 m exhibit lower CRF compared to those living in plain areas and internationally. Although exercise performance improves with age, CRF gradually decreases, which may be related to academic pressure or reduced exercise

Simultaneously, addressing the distinctive natural environment conditions and unique health status and disease characteristics among residents in the Northwest region, a Northwest Region Natural Population Cohort Study was established between 2017 and 2019 in Shaanxi, Gansu, Qinghai, Ningxia Hui Autonomous Region, and Xinjiang Uygur Autonomous Region. This study enrolled 117,644 multi-ethnic (including Han, Hui, Uyghur, Kazakh, and Tibetan) individuals aged between 35 and 74 years old, systematically collecting various exposure information at individual, environmental, and social levels and procuring a large number of peripheral blood samples. Over 900,000 biological samples of various types were stored in a standardized biobank. Currently, a combination of routine monitoring and active follow-ups is being used for long-term tracking observations. The average age of cohort members is 52.43 years, with women comprising 59.8%. There are differences in socioeconomic status and lifestyles among different ethnic groups, although they share some similarities in overall health status while displaying distinct traits. This Northwest Region Natural Population Cohort Research platform provides robust support for exploring the relationships between multiple factors such as environment, lifestyle, and genetics and major chronic diseases. It holds promise in providing key epidemiological evidence to elucidate the etiology of chronic diseases and their long-term health impacts among different ethnicities in the region. Ultimately, this could provide a scientific basis for formulating national chronic disease prevention and control strategies, not only for the Northwest region but also on a nationwide scale (Dang et al., 2023).

In the field of high-altitude illness research in China, Professor Shurong Han’s team from Shandong University conducted a retrospective cohort study focusing on maintenance workers of the Qinghai-Tibet Railway. This study enrolled 286 individuals working at high altitudes and through long-term follow-up and testing, found that during the observation period, heart morphological and functional abnormalities were present in 173 participants, with the most common being right atrial enlargement, left ventricular diastolic dysfunction, and tricuspid valve regurgitation. The findings further revealed that older workers engaged in work at extremely high altitudes were more prone to cardiac abnormalities compared to younger workers (under 20 years old) who worked at altitudes below 4,000 m. Overall, over 60% of the study subjects showed cardiac abnormalities after working at high altitude, primarily manifested as right heart enlargement and left ventricular diastolic dysfunction. Based on these results, the study emphasized the importance of employment age and workplace altitude as significant risk factors for cardiac abnormalities and recommended intensified regular cardiovascular health check-ups for workers at high altitudes (Han et al., 2020).

Another cohort study was carried out by the research team led by Dr. Renzheng Chen from the Second Affiliated Hospital of the Third Military Medical University (now Army Medical University), PLA Cardiovascular Disease Institute (Chen et al., 2021). They selected 72 subjects who ascended to the Lhasa region at an altitude of 4,100 m and used 24-h ambulatory blood pressure monitoring devices and echocardiography for detailed assessments. The study results showed that patients with Acute Mountain Sickness (AMS) had lower LA PP and Ea values, indicating a significant association between these baseline indicators of LA vascular relaxation and the occurrence of AMS, potentially explaining why subjects with higher LAPP experienced more severe headaches.

Professor Leping Zhang’s research group from Xinqiao Hospital, Army Medical University, employed a high-altitude field-based cohort study method to analyze the physiological changes in 83 young Chinese male subjects following acute exposure to high altitude (Zhang et al., 2019). The study reported that after acute high-altitude exposure, participants’ SAS (Social Support Rating Scale) scores, FSAS (Flight Anxiety Scale) scores, and heart rates significantly increased (*p* < 0.05), while oxygen saturation levels notably decreased (*p* < 0.05). Concurrently, there was a marked increase in systemic and cerebral blood flow velocities (*p* < 0.05), accompanied by elevated endothelin-1 and bradykinin levels (*p* < 0.05), as well as decreased concentrations of nitric oxide, prostaglandin E, and serotonin (*p* < 0.05). Based on these findings, the study concluded that left ventricular end-diastolic diameter (LVEDD) measured at sea level has some predictive value for AMS; moreover, changes in hemodynamics and psychological states such as anxiety and fatigue after high-altitude exposure may play a role in the development of AMS.

At the Sixth Medical Center of Traditional Chinese Medicine, PLA General Hospital 301, researchers led by Li Min conducted a field-based cohort study in high-altitude settings involving 80 participants to investigate the protective effects of Danshen-Qi-Jing Granules against acute mountain sickness (AMS) among rapid ascent personnel (Li et al., 2023). The treatment group was administered Danshen-Qi-Jing Granules, which primarily consist of herbs like Astragalus membranaceus, Salvia miltiorrhiza, and Polygonatum sibiricum, while the control group received Rhodiola rosea capsules. The study revealed that a continuous 14-day oral administration of the granules, initiated 7 days prior to ascending to high altitude, effectively reduced scores on the Syndrome Differentiation Quantitative Scoring Table for Traditional Chinese Medicine and improved clinical symptoms among the subjects. Additionally, the findings suggested that the granules might exert a protective role against AMS development through downregulating serum levels of TNF-α and IL-6.

At the Xinjiang Military Region General Hospital, a team led by Tang Zhi’ai conducted a comprehensive investigation into the health impacts of high-altitude environments on military personnel (Tang et al., 2020). The findings revealed clear correlations between physiological parameter alterations and both the duration of stay at high altitudes and the specific altitude levels occupied.

Researchers at the School of Medicine, Tibet University, including Bai Xueyazi, carried out an observational cohort study aimed at elucidating the variation in incidence rates of high-altitude illnesses under similar altitudes but contrasting microclimatic conditions (Baixue et al., 2023). The study observed a total of 251 migrants in two regions, A and B, on the Qinghai-Tibet Plateau, using a scoring system based on the international diagnostic criteria for chronic high-altitude illness. Results showed that Region A, characterized by significant seasonal temperature variations and harsh climate, had a significantly higher overall incidence rate of chronic high-altitude illnesses compared to the more temperate and lushly vegetated Region B. This indicated that aside from altitude, the microclimate environment of high-altitude regions also plays a pivotal role in influencing the prevalence of high-altitude illnesses among migrant populations.

A team led by Pasang Zhuoma from the same institution focused on hypoxia adaptation patterns in high-altitude areas (Gongga et al., 2014). Through studying blood pressure characteristics in 845 native Tibetan young adults living at elevations of 2,700, 3,650, and 4,700 m, they found that the prevalence of hypotension gradually increased with rising elevation, reaching 59.1%, 59.3%, and 76.9% respectively. This suggests that during long-term adaptation to high-altitude environments, the body does not rely on raising blood pressure as a means to enhance oxygen uptake. Furthermore, their survey on high-altitude maladaptation among employees and workers in Lhasa demonstrated that across respiratory, nervous, circulatory, and digestive systems, native Tibetans exhibit superior adaptive capacity than migrants. However, even among native Tibetans dwelling in high-altitude areas, there is still a notable degree of functional dysregulation resulting from incomplete adaptation (Tsring et al., 2017).

At the Plateau Medical Research Center of Tibet University, researcher Li Juan conducted a study on the relationship between pulmonary function and high-altitude adaptation among native Tibetan residents across different altitude gradients (Li et al., 2019; Fan et al., 2021). Through random sampling of 673 Tibetans from four distinct altitudinal regions—Yigong (2,227 m), Lhasa (3,600 m), Chamdo (4,500 m), and Purang County (5,018 m)—she primarily assessed their static lung ventilation function and basal physiological indicators. The results showed that Chamdo residents had significantly higher mean values for vital pulmonary function parameters such as FVC (forced vital capacity), PEF (peak expiratory flow rate), FEF25 (forced expiratory flow at 25% of FVC), and FET (forced expiratory time), suggesting that enhanced pulmonary ventilation is a key indicator of Tibetan populations' adaptation to high-altitude environments. However, when altitude increases to certain heights, such as in Purang County, this physiological regulatory capability might be suppressed, leading to a plateau or decline in pulmonary ventilation function.

A cohort study by Ciren Zhuoma and Penba’s team from the same center aimed to investigate the distribution of body mass index (BMI) across various altitudes in Tibet (Ciren et al., 2021). They selected a total of 872 native Tibetan residents living at 2,516, 3,660, 4,500, and 5,018 m as research subjects. The findings revealed that the detection rates of overweight and obesity initially rose and then fell with increasing altitude, reaching 50.4%, 56.2%, 46.7%, and 21.3%, respectively, thus uncovering for the first time the correlation between BMI and high-altitude adaptability: ascending altitude may affect BMI, resulting in relatively lower BMI among residents in high-altitude areas.

At the High Altitude Military Medical College under the Third Military Medical University, Gao Yuchi and Zhou Qiquan’s team analyzed blood biochemical indices of non-Qinghai resident examinees stationed in Xining (2,200 m). Data from 2008 to 2010 indicated that 35.6% of the population had abnormal triglyceride concentrations, 32.9% had elevated uric acid levels, and 10.9% had increased AST (aspartate transaminase) levels, indicating mild liver dysfunction (Yang et al., 2011). Subsequently, the team further validated the prevalence of hyperuricemia among male cadres residing at varying altitudes. By collecting blood biochemical data from 693 male cadre examinees living at 2,800, 3,400, and 4,500 m, they found that the incidence of hyperuricemia progressively increased with rising altitude, reaching 28.88%, 50.37%, and 69.86%, respectively. Concurrently, there was a notably high comorbidity rate (ranging from 1.69 to 2.21) between hyperuricemia and conditions like polycythemia, elevated serum creatinine levels, and overweight status. These research outcomes provide significant scientific foundations for delving into the pathogenesis of hyperuricemia in high-altitude dwellers, guiding clinical prevention and treatment measures, and improving acclimatization levels in these populations (Wang et al., 2014).

Researchers at the High Altitude Military Medical College of the Third Military Medical University, led by Yi Yuan Yue, conducted a thorough investigation into changes in cardiac function before and after rapid ascent to high altitude and their relationship with Acute Mountain Sickness (AMS) (Yi et al., 2017). They swiftly transported 42 healthy subjects from Chongqing at an elevation of 500 m to Lhasa at 3,658 m within 43 h. On the third day before and after arrival, a series of assessments were made on the subjects' heart rate, blood pressure, oxygen saturation levels, echocardiograms, and Lake Louise Score. The study revealed that following the rapid ascent to high altitude, the subjects experienced elevated pulmonary artery systolic pressure (PASP) and mean pulmonary artery pressure (MPAP), along with enhanced left ventricular contractility. The incidence rate of acute mountain sickness was recorded as 35.7%, and notably, those in the AMS group had higher PASP values compared to the non-AMS group. Additionally, there was a negative correlation observed between cardiac output and AMS scores, suggesting that individuals with higher pulmonary artery systolic pressure and lower cardiac output at low altitudes are more prone to develop AMS upon entering high-altitude environments, thereby providing new insights for identifying and screening susceptible populations for AMS. Furthermore, the research team also focused on the correlation between sleep quality and chronic high-altitude diseases (Zhou et al., 2017). A total of 1,543 participants were enrolled, including 956 stationed at altitudes ranging from 3,200 to 5,400 m for over 6 months, while the control group consisted of 587 participants stationed in plains areas with elevations between 150 and 1,700 m. Utilizing the Pittsburgh Sleep Quality Index (PSQI) and Chronic Mountain Sickness Symptoms Questionnaire (CMS), it was found that the sleep quality of the high-altitude group was significantly lower than the plains group. In both healthy subgroups and those with CMS, the PSQI total scores were substantially higher in the high-altitude group, and there was a moderate positive correlation (r = 0.2) between the PSQI total score and the CMS score. These findings underscored the significant detrimental effect of high-altitude environments on sleep quality and its strong association with the occurrence of chronic high-altitude diseases. Therefore, it is crucial to simultaneously improve sleep quality and strengthen preventive and therapeutic measures for chronic high-altitude diseases, which corroborates the conclusions drawn by He Feng’s research at the General Hospital of the Tibet Military Region (He et al., 2013).

A longitudinal cohort study by Yuan Zhi Min et al. from Ali District People’s Hospital of Tibet investigated the impact of high altitude on hematological indices in Tibetan and Han populations (Yuan et al., 2023). This retrospective analysis, a first of its kind, monitored changes in blood indices over 3 years for low-altitude Han individuals who migrated to extreme heights, contrasting them with native Tibetans. The study involved 114 Tibetans and 93 Han subjects living at high altitudes for over 2 years. Results showed that migrating Han individuals experienced increased platelet, white blood cell, and red blood cell counts, suggesting sustained hematopoietic system stress to adapt to the environment. Additionally, this group had elevated TBIL, DBIL, and triglyceride levels, indicating heightened liver metabolic burden and potentially increased cardiovascular risk under hypoxic conditions. These findings underscore unique stress responses and adaptive differences in hematological profiles and liver-kidney functions between Han immigrants and native Tibetans, thereby contributing valuable data for future research into high-altitude population health.

At Fudan University’s School of Life Sciences, the research team led by Wang Jiucun conducted an observational cohort study investigating physiological, hematological, and biochemical risk factors associated with high-altitude headache (HAH) following acute high-altitude exposure (Wang et al., 2018). The study enrolled 318 Han Chinese male participants who underwent a rapid ascent to an altitude of 3,700 m within 72 h via train travel from an elevation of 50 m, simulating a scenario of rapid ascent to high altitudes. The study findings revealed that HAH incidence reached a striking 74.84% among the subjects, emphasizing HAH as one of the most prevalent symptoms in acute mountain sickness. Notably, significant physiological changes were observed in these individuals upon transitioning from lowland to high-altitude conditions; oxygen saturation levels dropped from approximately 98% at sea level to 88% post-ascent, and hematological parameters such as red blood cell count, mean corpuscular volume, and hemoglobin concentrations, along with liver and kidney function markers like alanine transaminase and creatinine levels, all exhibited marked elevations. Through subgroup analyses comparing HAH patients with non-HAH participants, the research team substantiated that decreased oxygen saturation and altered heart rate serve as independent risk factors for the development of HAH. Moreover, they made the novel finding that elevated blood urea nitrogen (BUN) levels also constitute an independent risk factor for HAH. These discoveries contribute significantly to the understanding of the pathophysiological mechanisms underlying headache occurrence during acute high-altitude reactions and are pivotal for devising preventive and therapeutic strategies for high-altitude illnesses.

At the Institute of Psychology, Chinese Academy of Sciences, Liu Zhengkui’s team conducted a large-scale investigation into depression and its risk factors among the Tibetan population (Wang et al., 2019). The study involved 24,141 adult residents from Yushu Tibetan Autonomous Prefecture in Qinghai Province. Employing the Center for Epidemiologic Studies Depression Scale (CES-D), they discovered that the hypobaric hypoxic environment prevalent in high-altitude regions is intricately linked to individuals’ mental state, fatigue levels, and neural activity, thereby exacerbating negative emotions and contributing to significant mood disorders. Their findings revealed a striking prevalence rate of depression at 28.6% in high-altitude areas, with a positive correlation between depression incidence and altitude. Furthermore, gender, age, income level, educational attainment, and self-perception were also identified as relevant risk factors for depression.

Wang et al. (2023) from the Department of Psychiatry at the Medical College of the National Defense Medical Center explored the relationship between Acute Mountain Sickness (AMS) and the risk of psychiatric disorders in Taiwan. After conducting a 16-year follow-up study on 127 AMS patients and 1,270 controls, and analyzing the data using the Fine-Gray model, they found that AMS sufferers had a relatively higher likelihood of developing mental illnesses. Specifically, AMS was associated with increased risks of anxiety disorders, depression, bipolar disorder, sleep disorders, post-traumatic stress disorder/acute stress disorder, schizophrenia, and other substance-related disorders.

Ma et al. (2018) approached the issue from a spatial epidemiological perspective by presenting the first report on the spatial distribution and changes of congenital heart disease (CHD) among children living on the Qinghai-Tibet Plateau. They analyzed epidemiological data from 288,066 children aged 4–18 years old in this region, categorizing CHD prevalence according to sex, subtypes of CHD, ethnicity, and altitude. The study disclosed an overall CHD prevalence rate of 5.8% among children, with girls exhibiting significantly higher rates than boys; atrial septal defect was the most common subtype. Among different ethnic groups, Mongolians had the highest prevalence, followed by Tibetans, Han, and Hui, potentially reflecting distinct hypoxia adaptation genes or genetic characteristics within these populations. Notably, CHD prevalence escalated with increasing altitude, reaching its peak in Yushu Tibetan Autonomous Prefecture at 4,000 m above sea level. This research uncovered a pronounced spatial clustering pattern of CHD in the Qinghai-Tibet Plateau, emphasizing the substantial influence of environmental factors on CHD prevalence. It thus provides crucial reference information for formulating preventive strategies and reducing CHD incidence in high-altitude areas in clinical practice.

Researchers from the China Institute of Sport Science and the Tibet Institute of Sports Science, including Fan Chaoqun and Nie Mingjian among others, conducted a cross-sectional study on cardiorespiratory fitness (CRF) among adolescents living in high-altitude regions (Fan et al., 2021). They randomly sampled nearly 2,000 students aged between 9 and 18 years from a school located at an elevation of 3,500 m in Tibet, employing the 20-m shuttle run test (20mSRT) as a measure of aerobic endurance capacity. This assessment indirectly reflected cardiovascular function by quantifying completed laps and predicting peak oxygen uptake. The research findings indicated that adolescents residing in altitudes above 3,000 m displayed a generally lower CRF compared to their peers in lowland areas such as Shanghai and other international locations. While there was some improvement in physical performance with age, CRF exhibited a declining trend over time in this population. This phenomenon might be associated with increased academic pressure and reduced exercise time experienced by adolescents in the high-altitude environment.

### 2.2 International advances in cohort studies

Dr. Charles B. Duke from the Department of Emergency Medicine at Yale School of Medicine led a team that conducted an observational cohort study aimed at investigating the association between hypertension and Acute Mountain Sickness (AMS) among trekkers in the Himalayas of Nepal. The study enrolled 672 participants who engaged in hiking activities at an altitude of 2,860 m, including 60 individuals with a history of hypertension. By recording Lake Louise Scores and monitoring blood pressure changes at altitudes of 2,860, 3,400, and 4,300 m, researchers primarily focused on AMS incidence rates. The results showed that AMS was prevalent among trekkers in Nepal, but no correlation was observed between blood pressure measured at 3,400 m and AMS incidence. Notably, a history of hypertension seemed to be related to a potentially lower risk of developing AMS (Duke et al., 2020).

Researchers at the Department of Medicine, Armed Forces Medical College in Pune, India, led by Dr. Virendra Narain, carried out a prospective longitudinal study to delve into the epidemiology and pathophysiology of thrombosis formation in lowland residents adapting to high-altitude environments (Nair et al., 2022). Initially, they screened 960 healthy male subjects at sea level, with 750 subsequently transported to regions above 15,000 feet (4,472 m). The study revealed that the high-altitude environment induced a prothrombotic state characterized by decreased natural anticoagulant activity, fibrinolysis suppression, endothelial cell activation, platelet activation, and elevated inflammatory markers. The findings demonstrated that, within this group of healthy subjects, the incidence rate of clinically significant thrombotic events (predominantly venous over arterial thromboses) was notably higher at elevations exceeding 15,000 feet (4,572 m) compared to reported values near sea level. Thus, it was concluded that altitudes above 15,000 feet (4,572 m) may serve as an independent risk factor for thrombosis formation, even among otherwise healthy individuals.

A research team led by Dr. Michel Claes, MD, from the Institute of Tropical Medicine (ITM), Belgium, conducted a prospective cohort study examining the incidence of severe altitude illness symptoms and their predictive factors among Kilimanjaro trekkers (Croughs et al., 2022). The study included 1,237 leisure hikers and 266 porters or guides. The data showed an 8.6% incidence rate of severe altitude illness symptoms among leisure hikers, whereas the proportion was only 1.5% for porters and guides. About 1.1% of leisure hikers required hospitalization due to Severe Acute Mountain Sickness (SAI). Factors such as a history of SAI, youth, failure to reach the summit, lack of clear ascent recommendations were found to be associated with a higher likelihood of experiencing severe altitude illness symptoms. In contrast, the absence of severe symptoms, the use of acetazolamide prophylaxis, ascending to higher altitudes during daylight hours, younger age, and longer climbing durations were all significantly correlated with successful summiting. The study emphasized the critical importance of avoiding further ascent when mild symptoms are present and descending immediately upon the onset of severe symptoms. Based on these findings, the study concluded that SAI incidence is alarmingly high among Kilimanjaro trekkers, emphasizing the need for travel health advisors to underscore the importance of not ascending until mild symptoms have subsided and the necessity for immediate descent when severe symptoms arise.

At the Department of Sports Science at the University of Innsbruck, Austria, Professor Martin Faulhaber and his team conducted a prospective cohort study to assess whether resting arterial oxygen saturation (SaO_2_) and respiratory rate could serve as effective predictors for the development of Acute Mountain Sickness (AMS) (Faulhaber et al., 2014). The research involved placing 55 subjects in an environment simulating an altitude of 4,500 m, achieved under normobaric hypoxia conditions with a fractional inspired oxygen concentration (FiO2) of 12.5%. After just 30 min of exposure to the low-oxygen environment, researchers measured cardiovascular and pulmonary function parameters, including oxygen saturation levels, blood lactate concentrations, and blood pressure in the participants. Subsequently, AMS symptoms were recorded using the Lake Louise Scoring System at intervals of 3, 6, 9, and 12 h post-exposure. Through multivariate logistic regression analysis, the study explored whether incorporating additional physiological measurements at rest could enhance predictive accuracy for AMS onset. The results demonstrated that, after only 30 min of hypoxic exposure, non-invasive measurement of resting SaO_2_ was both convenient and sufficiently sensitive to identify individuals susceptible to high-altitude reactions. Moreover, incorporating respiratory rate as a variable further improved the accuracy and predictive power for identifying those at risk of developing AMS. This study provides new practical tools and theoretical support for the prevention and management of acute mountain sickness.

At the Institute of Social and Preventive Medicine at the University of Zurich, Professor Susi Klimmer’s research team conducted a prospective cohort study aimed at investigating symptoms, incidence rates, and associated risk factors for Acute Mountain Sickness (AMS) among family members rapidly ascending to 3,450 m (Kriemler et al., 2014). The study included 87 children, 70 adolescents, and 155 parents, totalling 312 participants who were assessed using the Lake Louise Score (LLS) system for AMS symptom severity at two time points—8–10 h and 20–24 h post-ascent. They also monitored pain sensitivity and oxygen saturation (SO2), as well as analyzed clustering of AMS symptoms among family members. The findings revealed that on the first day following rapid ascent to 3,450 m, children had a lower prevalence of AMS compared to adolescents and adults; however, by the second day, this difference was no longer evident. This suggests that despite potential risk factors such as pain sensitivity and possible genetic influences, children can tolerate entering high-altitude regions like 3,500 m as safely as adolescents and adults, at least in the short term. This study provides valuable scientific insights into the differing abilities of people across different age groups to adapt to high-altitude environments. For details of the aforementioned studies, see Table 3.

**TABLE 3:** International advances in cohort studies.

Team	Time	Participant	Altitude	Conclusion
Dr. Charles B. Duke from the Department of Emergency Medicine at Yale School of Medicine led a team Duke et al. (2020)	2014	672 participants who engaged in hiking activities at an altitude of 2,860 m	Hiking activities at an altitude of 2,860–4,300 m	AMS was prevalent among trekkers in Nepal, but no correlation was observed between blood pressure measured at 3,400 m and AMS incidence. Notably, a history of hypertension seemed to be related to a potentially lower risk of developing AMS
Researchers from the Medical Department of the Pune Armed Forces School of Medicine, led by Dr. Virendra Narain Nair et al. (2022)	2012–2014	A total of 960 healthy male subjects were screened in the plains, of which 750 ascended, to altitudes above 15,000 ft (4,472 m)	Altitude: 4,472 m	In healthy populations, the incidence rate of clinically significant thrombotic events (predominantly venous over arterial thromboses) was notably higher at elevations exceeding 15,000 feet (4,572 m) compared to reported values near
research team led by Dr. Michel Claes, MD, from the Institute of Tropical Medicine (ITM), Belgium Croughs et al. (2022)	2019–2020	1,237 leisure hikers and 266 porters or guides	Altitude: 5,895 m	Factors such as a history of SAI, youth, failure to reach the summit, lack of clear ascent recommendations were found to be associated with a higher likelihood of experiencing severe altitude illness symptoms. In contrast, the absence of severe symptoms, the use of acetazolamide prophylaxis, ascending to higher altitudes during daylight hours, younger age, and longer climbing durations were all significantly correlated with successful summiting
Professor Martin Faulhaber and his team at the Department of Sports Science, University of Innsbruck, Austria Faulhaber et al. (2014)	2013	55 healthy adults aged 18–35	Altitude: 4,500 m, achieved under normobaric hypoxia conditions with a fractional inspired oxygen concentration (FiO2) of 12.5%	After only 30 min of hypoxic exposure, non-invasive measurement of resting SaO_2_ was both convenient and sufficiently sensitive to identify individuals susceptible to high-altitude reactions. Moreover, incorporating respiratory rate as a variable further improved the accuracy and predictive power for identifying those at risk of developing AMS.
The Institute of Social and Preventive Medicine at the University of Zurich, Professor Susi Klimmer’s research team Kriemler et al. (2014)	2005–2009	87 children, 70 adolescents, and 155 parents, totalling 312 participants	Rapidly rising to an altitude of 3,450 m	Despite potential risk factors such as pain sensitivity and possible genetic influences, children can tolerate entering high-altitude regions like 3,500 m as safely as adolescents and adults, at least in the short term. This study provides valuable scientific insights into the differing abilities of people across different age groups to adapt to high-altitude environments

The incidence and influencing factors of high-altitude illnesses in populations have been subject to several pivotal breakthroughs through cohort studies both domestically and internationally. The research has primarily focused on three central domains:

(1) Identification of risk factors associated with the onset of high-altitude illnesses: Large-scale epidemiological surveys and in-depth data analyses have demonstrated significant associations between physiological parameters such as age, sex, body mass index, and pulmonary function, and an individual’s adaptability to high-altitude environments, as well as the incidence and severity of high-altitude illnesses.
(2) Exploration of the genetic underpinnings of high-altitude illnesses: Rigorous genetic research has uncovered a number of genes, including EGLN1 and EPAS1, that show strong links with susceptibility and tolerance to high-altitude illnesses, thereby providing crucial insights into the genetic regulatory mechanisms underlying adaptation to high altitude.
(3) Evaluation of the efficacy of preventive and intervention strategies: Systematic comparisons of various preventive and interventional measures have substantiated that pharmacological preconditioning with agents like acetazolamide and targeted acclimatization training can effectively reduce the incidence of high-altitude illnesses and mitigate the severity of their clinical manifestations.

In summary, population-based cohort studies targeting high-altitude illnesses are irreplaceably significant for delving into the pathophysiology of the disease and scientifically assessing the practical effects of diverse preventive measures. These studies have thus robustly guided efforts aimed at improving health outcomes and ensuring safety for residents living in high-altitude regions.

## 3 Research on predictive indicators and model construction for AMS

**FIGURE 3:**
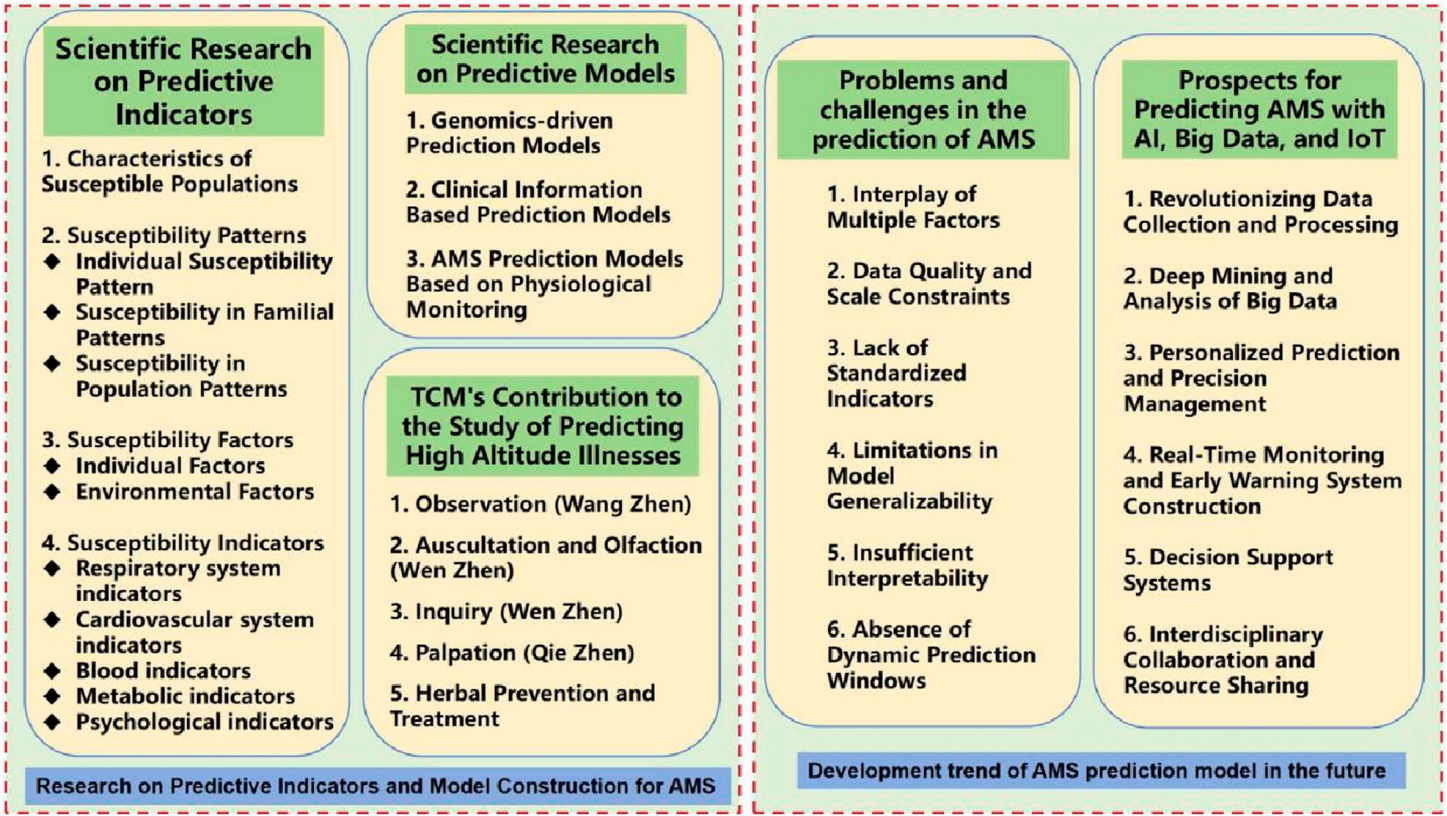
Research on predictive indicators, model construction for AMS and its future development trend.

Research on predictive models for high altitude illnesses centers around the development of mathematical models and statistical analyses to accurately forecast an individual’s probability of developing AMS in high-altitude environments based on specific attributes and physiological markers. These prediction models are designed to identify susceptible populations by deeply exploring various patterns of susceptibility, related risk factors, and the impact of specific indicators on AMS onset. The aim is to uncover highly specific vulnerability parameters that can effectively screen out high-risk groups, enabling precise allocation of preventive measures, mitigating potential risks, and reducing incidence rates. The overall framework is illustrated in Figure 3.

### 3.1 Scientific research on predictive indicators

#### 3.1.1 Characteristics of susceptible populations

AMS susceptible populations refer to individuals who are more likely to develop illness or exhibit more severe symptoms under high-altitude conditions. Compared to the general population, these individuals demonstrate weaker adaptability and more pronounced, serious symptoms in response to high altitude (Wei, 2023). Susceptibility can be broadly or narrowly defined: Broadly, it encompasses any group with increased disease risk upon entering high altitudes, such as those with chronic respiratory or cardiovascular diseases, or individuals with known triggering factors. In a narrower sense, it specifically refers to individuals without prior medical history or known triggers who suddenly develop AMS upon reaching high altitudes (Niu et al., 2012). Currently, screening for this narrow subset of susceptible individuals represents a critical challenge in AMS prediction research. Researchers primarily assess changes in human functional status, tissue factors, and protein expression differences before and after exposure to high altitudes, with some scholars beginning to explore genetic screenings for high-altitude susceptibility, despite numerous challenges. Genetic screenings could potentially hold significant promise for the future (Zhang and Zhang, 2010).

It is worth noting that there is no uniform standard or clear boundary defining AMS susceptible populations. This is due to the complex interplay of numerous factors influencing AMS susceptibility, including individual age, health status, genetic background, preconditioning through adaptive training, among intrinsic conditions, as well as external environmental factors like altitude level, duration of exposure, and rate of ascent. Therefore, the concept of a susceptible population for acute mountain sickness is relative, characterized by a degree of subjectivity and variability. Based on different research practices and outcomes, the identification and definition of susceptible populations can be achieved by considering multiple dimensions of data, including personal information, health status, genetic factors, and adaptive capacity (Bartsch et al., 2004; Luks et al., 2010; Luks et al., 2017).

#### 3.1.2 Susceptibility patterns

The susceptibility pattern for AMS refers to the regularized expression that describes an individual’s tendency to develop AMS under high-altitude conditions. This pattern encompasses a multitude of factors, including physiological characteristics, genetic background, and environmental conditions, which are crucial in predicting and intervening against the occurrence of high-altitude illnesses (Zhou and Hu, 2013).

##### 3.1.2.1 AMS individual susceptibility pattern

Researchers such as Bartsch et al. (2004) and Wagner et al. (2006) have found that individuals with a history of AMS are more likely to experience AMS upon re-entry into high-altitude environments compared to those without a history, indicating a distinct susceptibility trait in AMS. Further, Schneider et al. (2002) tracked 847 climbers ascending to Capanna Margherita (altitude 4,559 m), revealing that AMS history is one of the critical determinants influencing a climber’s ability to successfully summit. In this study, among the 235 climbers with AMS history who did not reach the summit, they experienced AMS episodes two to three times more frequently than the average for successful summiteers, suggesting that AMS history is the second most significant risk factor for severe AMS after residence altitude. At the Cardiovascular Disease Institute of the Chinese People’s Liberation Army, Ke et al. (2021) identified a novel echocardiographic predictor associated with AMS onset through analysis of new ultrasound parameters. The research demonstrated that participants with relatively weaker left ventricular longitudinal systolic function at sea level were at a higher risk of developing AMS following acute high-altitude exposure. This may be related to differing degrees of systolic and diastolic functional changes in both ventricles under acute high-altitude conditions, contributing to inter-individual variations in AMS susceptibility. Moreover, due to substantial unexplained inter-individual variability in AMS severity, non-targeted metabolomics analyses have been widely employed to identify new disease-sensitivity biomarkers and elucidate biological pathways linking environmental exposures to health outcomes. Dr. Sibomana I from the United States (Sibomana et al., 2021), along with colleagues, used a non-targeted metabolomics approach based on nuclear magnetic resonance (NMR) to investigate urine metabolite changes associated with AMS severity during high-altitude stays. Their study involved 17 healthy male participants, and pathway analysis of differentially expressed metabolites revealed their direct or indirect involvement in energy metabolism processes. These findings suggest that changes in energy metabolism prior to entering high-altitude environments might increase an individual’s sensitivity to AMS. Such discoveries contribute to the development of non-invasive screening methods to identify AMS susceptibility before high-altitude sojourns.

##### 3.1.2.2 AMS susceptibility in familial patterns

Genetic diseases often show higher incidence rates within certain families. Examples include cystic fibrosis, sickle cell anemia, Huntington’s disease, familial hypercholesterolemia, and autosomal dominant polycystic kidney disease, which have a higher incidence in certain families (Abdelnour et al., 2021; Reiterová and Tesar, 2022; Abifadel and Robinson, 2023) HAPE (High Altitude Pulmonary Edema) is one such genetically influenced disease that interacts with environmental factors. In recent years, multiple domestic and international studies have revealed a clear genetic predisposition for HAPE among family members. For instance, Eichstaedt et al. analyzed PAH candidate genes in HAPE-prone families and mountaineers by next-generation sequencing technology, and identified the association between PAH-related pathogenic mutations in JAK2 gene and HAPE for the first time, and found other pathogenic variants in some HAPE-prone mountaineers, suggesting that genetic predisposition is closely related to PAH signaling pathways (Eichstaedt et al., 2020). Through case-control analysis of Chinese Han population, Si L et al. found that specific polymorphisms of CYP4F2 gene (rs3093193 and rs12459936) were significantly associated with susceptibility to high altitude pulmonary edema (HAPE). rs3093193 may decrease the risk of HAPE, while rs12459936 may increase the risk of HAPE, and these association effects are influenced by age and sex (Si et al., 2023) Luo et al. found for the first time that mtDNA 3397A/G and 3552T/A variants were associated with HAPE susceptibility in Han population by comparing mitochondrial DNA polymorphisms in patients with high altitude pulmonary edema and healthy controls, especially the frequencies of 3397G and 3552A genotypes were significantly increased in HAPE patients. It is suggested that mitochondrial genetic factors may be involved in regulating the pathogenesis of HAPE (Luo et al., 2012).Guoen Jin et al. observed the incidence of acute high-altitude disease in different regions and found that its pathogenesis is related to certain mutation forms of hypoxia related genes, the number and function of protein coding genes, such as HIF1A, EPO, EDN1, NOS3, and also related to human leukocyte antigen (HLA). Azad P’s group found through whole genome sequence analysis of CMS and non CMS subjects in the Andes region that sentrin-specific protease 1(SENP1) is the main regulatory factor for red blood cell generation in the CMS population, and these SNPs exhibit significant differences in allele frequencies (differential SNPs) between CMS and non CMS individuals.

##### 3.1.2.3 AMS susceptibility in population patterns

Over millennia, human populations in areas like the Andes, the Tibetan Plateau, and the East African Highlands have evolved through natural selection, resulting in some groups acquiring genetic adaptations against high-altitude illnesses. Wu et al. (2005, 2009) studies from 2005 to 2009 suggested that compared to Han Chinese who are native to lowlands, Tibetans, as well as Chinese Tibetan workers constructing along the Qinghai-Tibet Railway, have significantly lower incidences of acute mountain sickness, possibly reflecting a genetic adaptive advantage in Tibetans. MacInnis and team also found in a large-scale prospective field study that individuals of Tibetan descent were at lower risk of AMS compared to Indian-White populations (MacInnis et al., 2013). Similarly, Wu’s reports indicate that Tibetans and Sherpas are less prone to AMS than their Han Chinese and Japanese counterparts during trekking activities (Wu et al., 2005). However, in these studies, due to the challenge of fully excluding potential confounding effects of environmental differences such as health status, dietary habits, and cultural practices, interpretations of the conclusions should be approached with caution.

In summary, the susceptibility patterns for Acute Mountain Sickness embody a patterned representation of an individual’s characteristics that render them more likely to develop the condition. These patterns provide essential theoretical underpinnings for understanding the various factors determining individual susceptibility, assessing risk levels, formulating personalized management strategies, and predicting and intervening in high-altitude illnesses.

#### 3.1.3 Susceptibility factors

AMS susceptibility factors are the various conditions in high-altitude environments that affect an individual’s likelihood of developing AMS. These factors can be broadly classified into individual and environmental factors.

(1) Individual Factors:

Age: While early studies suggested a possible link between age and AMS, a meta-analysis conducted by researchers at the Military Medical Geography Department of Chongqing Medical University led by Wu et al. (2018), involving 17 studies and a total of 4,824 participants (1,810 with AMS symptoms and 3,014 without, ranging from 10 to 76 years old), found no significant association between age and AMS incidence.

Health status: Individuals with specific health issues or chronic diseases may be more susceptible to altitude illnesses. Conditions such as cardiovascular diseases, respiratory disorders, multi-systemic illnesses like anemia, and pregnant women exhibit increased sensitivity to hypoxic environments (Hackett et al., 1976; Wu et al., 2014).

Innate characteristics: Genetic factors also play a role in altitude illness susceptibility. Multiple studies have identified genes including HIF-1, EPO, VEGF, EGLN1, and EPAS1 as being associated with susceptibility and adaptability to altitude illnesses (Gong et al., 2012). For example, Boyd C M’s research (Boyd, 2010) highlighted the impact of genetic factors on physiological variables, particularly those encoding angiotensin-converting enzyme (ACE) genes and endothelial nitric oxide synthase (eNOS), which were associated with AMS, suggesting these two genes as potential genetic biomarkers for AMS.

Individual adaptability: An individual’s history of exposure to high altitude and their level of acclimatization training significantly influence their susceptibility. Those who lack sufficient acclimatization or prolonged exposure to high altitudes are more prone to altitude illnesses (Li et al., 2011).

Psychological factors: Psychological states, such as anxiety, stress, and fear, may increase an individual’s sensitivity to high-altitude environments and impact normal functioning of respiratory and circulatory systems. Research by Boos et al. (2018) indicates that baseline anxiety levels in low-altitude dwellers independently predict future severe AMS episodes; state anxiety in hypertensive patients is also independently associated with AMS occurrence and severity.

(2) Environmental Factors:

Altitude: As altitude increases, oxygen pressure and oxygen content decrease correspondingly, increasing the risk of altitude-related illnesses. Beidleman BA et al. from the United States Army Research Institute of Environmental Medicine (Beidleman et al., 2013) developed a model that quantitatively estimated AMS risks at different altitudes and time points. By analyzing AMS scores from 308 subjects across 1,292 data points, the study found that AMS severity nearly doubled for every 1,000-m increase in altitude and peaked around 18–22 h of exposure, reverting to initial levels by 48 h of exposure, a pattern unaffected by gender or activity level. Although males had an increased risk of AMS compared to females, the difference was not statistically significant (*p* = 0.10).

Exposure time and rate: The longer an individual is exposed to high altitude and the faster they ascend, the higher the probability of experiencing altitude illness (Xue et al., 2011). Harrison et al. (2013) in the Antarctic Study of Altitude Physiology (ASAP) discovered that fluid regulation and inflammatory responses within the body are key factors contributing to AMS in adults following passive rapid ascents to high altitudes, even when controlling for other factors like physical exertion levels, ascent rates and magnitudes, and acetazolamide use.

Temperature and humidity: Extreme cold or hot environmental conditions might also enhance an individual’s sensitivity to altitude illnesses (Aoki and Robinson, 1971; Loeppky et al., 2003).

Climate change and weather conditions: Climate change, barometric changes, and different weather conditions could also influence an individual’s susceptibility to acute mountain sickness (Wang et al., 2010).

It is crucial to note that all these susceptibility factors interact and vary among individuals. Thus, when assessing an individual’s AMS susceptibility, it is essential to consider multiple variables holistically and employ scientific methods for personalized assessment and management.

#### 3.1.4 Susceptibility indicators

Acute altitude sickness susceptibility assessment indicators are physiological characteristics or parameters used to determine an individual’s susceptibility to developing acute altitude sickness in high altitude environments. These indicators are primarily based on the individual’s physiological status, adaptability, and environmental conditions. The following are potential indicators of acute altitude sickness susceptibility:

(1) Respiratory system indicators: These involve respiratory frequency, lung function, and oxygenation capacity. In high altitude environments, the adaptability of the respiratory system significantly impacts hypoxia tolerance and the risk of altitude sickness. For example, Wang et al. (2018) found that the prophylactic use of COM (a combination of ipratropium bromide and salbutamol sulfate) can reduce the incidence of acute altitude sickness (AMS) and LLS in Chinese male youths exposed to high altitudes for 72 h, but it cannot fully prevent AMS within 72 h. M Faulhaber et al. (Croughs et al., 2022) conducted a prospective cohort study that found resting arterial oxygen saturation and respiratory rate to be effective predictors of Acute Mountain Sickness (AMS). The study assessed the predictive value of arterial oxygen saturation (SaO_2_) after 30 min of hypoxic exposure for subsequent AMS occurrence and investigated whether additional resting cardiorespiratory parameters could improve AMS prediction accuracy. The results showed that non-invasive measurement of SaO_2_ after 30 min of hypoxic exposure was simple, practical, and capable of identifying AMS-susceptible individuals with high sensitivity; moreover, combining it with respiratory rate measurements further improved the success rate in predicting AMS. Despite limited data and inconsistent results on the association between lung function tests and AMS, Small et al. from Stanford University School of Medicine (Small et al., 2021) explored the predictive capacity of pulmonary function tests through a double-blind, prospective observational study. They compared acetazolamide, budesonide, and placebo in preventing AMS in a randomized controlled trial at White Mountain, California. Researchers measured lung volume indicators such as FEV1 (forced expiratory volume in one second), FVC (forced vital capacity), and peak expiratory flow rates, and found no statistically significant association between these indices and AMS development at baseline altitude (1,250 m) or after ascent to 3,810 m. Therefore, low-altitude lung function tests do not accurately predict AMS onset and are not recommended for risk stratification.
(2) Cardiovascular system indicators: These include heart rate, blood pressure, heart rate variability (HRV), and cardiac load. The adaptability of the cardiovascular system plays a crucial role in altitude sickness susceptibility as it needs to adapt to the hypoxic and high-load conditions of high altitudes (Chen et al., 2021).For example, Huang Xianhao’s team (Han et al., 2020), on the other hand, focused on the predictive role of low-altitude heart rate variability (HRV) in acute mountain reactions among trekkers at high altitudes. In a prospective study, they monitored HRV (including total variance, HF, and HF%) in 32 trekkers heading to Namche Bazaar (altitude 3,440 m) in Nepal for a 12-day journey. Results showed that trekkers experiencing AMS symptoms had significantly lower HRV spectral analysis parameters like total variance, HF, and HF%. It was proposed that at low altitudes, HF% < 20% (nu) or LF:HF ratio > 1.3 might be critical indicators for predicting AMS symptoms at higher altitudes. This study delved into the changes in HRV spectral components and their correlations in subjects with or without acute mountain sickness (AMS) under different altitude conditions. Sutherland et al. (2017) from Queen Alexandra Hospital in Portsmouth, United Kingdom, also confirmed the advantage of Heart Rate Variability (HRV) in early AMS prediction. In their research expedition in the Himalayas of Nepal at elevations over 5,000 m, they recorded SpO2 and HRV parameters in healthy volunteers under normoxic and simulated high-altitude hypoxic conditions and collected Lake Louise Scores daily at 3,841 m to assess AMS symptoms. The findings indicated that HRV measurements taken before ascent under normal oxygen conditions had superior diagnostic accuracy for AMS compared to all HRV measurements and peripheral oxygen saturation monitoring during hypoxia, thus affirming the predictive role of HRV assessment both before and during expeditions for AMS susceptibility at high altitudes, with its diagnostic accuracy surpassing SpO2 monitoring.
(3) Blood indicators: These include hemoglobin levels, red blood cell counts, and plasma viscosity. These indicators are associated with the oxygen transport capacity and hemorheological properties of blood and may influence an individual’s susceptibility to altitude sickness (Dyer et al., 2008).
(4) Metabolic indicators: These include blood glucose levels and lactate levels. In high altitude environments, an individual’s metabolic status may affect energy supply and hypoxia tolerance (Bailey et al., 2003).
(5) Psychological indicators: These include measures such as the Eysenck Personality Questionnaire, the Self-rating Anxiety Scale, the Self-rating Depression Scale, and the Symptom Checklist-90. These non-invasive indicators have applications in predicting acute altitude sickness (AMS) (Guo et al., 2006; Zhang et al., 2020).

### 3.2 Scientific research on predictive models

In the construction of predictive models for assessing an individual’s susceptibility to Acute Mountain Sickness (AMS), it is imperative to systematically integrate a multitude of indicator parameters and employ classical statistical frameworks alongside cutting-edge artificial intelligence techniques, such as machine learning algorithms and deep learning architectures, to forge highly accurate predictive models. Concurrently, these models should be underpinned by rigorous scientific evaluation methods and personalized risk management strategies, facilitating in-depth predictive analyses. The predictive models established in current research can be broadly categorized into three classes based on the distinct source data features employed during model development.

#### 3.2.1 Genomics-driven prediction models

This category emphasizes the use of genetic information from genomic assays to uncover potential genetic biomarkers that inform the creation of mathematical models capable of forecasting an individual’s predisposition to acute high-altitude sensitivity. By parsing genomic data, these models aim to elucidate the role of genetics in determining AMS susceptibility.

MicroRNAs (miRNAs) have been established as potential sensitive and specific biomarkers for a variety of diseases, presenting extensive application prospects. Researchers from the High-altitude Medical Equipment Research Institute at the Third Military Medical University’s Academy of Military Sciences, led by Liu et al. (2017), conducted a study involving 109 healthy adults. Prior to their ascent to high altitude, blood samples were collected from these individuals. Utilizing microarray technology and quantitative reverse transcription polymerase chain reaction (qRT-PCR), they examined circulating miRNA expression levels in an attempt to identify miRNAs predictive of AMS susceptibility before exposure to high altitude. Through multivariate logistic regression analysis, it was discovered that a unique signature composed of miR-369-3p, miR-449b-3p, and miR-136-3p showed significant discriminative power between AMS and non-AMS cases (Area Under the Curve or AUC was 0.986, with 95% Confidence Interval or CI of 0.970–1.000, *p* < 0.001, positive likelihood ratio LR+ of 14.21, and negative likelihood ratio LR− of 0.08). This signature demonstrated a sensitivity of 92.68% and specificity of 93.48% for distinguishing AMS from non-AMS cases. Therefore, this study is the first to propose that the distinct expression profile of these three circulating miRNAs could serve as potent biomarkers for predicting AMS susceptibility prior to entering high-altitude environments.

Simultaneously, untargeted metabolomics techniques are being widely used to discover novel biomarkers for disease susceptibility and to delve into biological pathways connecting environmental exposures with health outcomes. At the Henry Jackson Foundation for the Advancement of Military Medicine, Sibomana et al. (2021) investigated whether urinary metabolites could act as effective predictors of AMS severity. Their study employed an untargeted metabolomics approach based on Nuclear Magnetic Resonance (NMR) spectroscopy to reveal changes in urine metabolites associated with AMS severity during high-altitude stays. The results showed that creatine and acetylcarnitine levels were elevated, while hypoxanthine and taurine levels were reduced in the AMS group compared to the no-AMS control group. Additionally, in the moderate symptom (SL) group, two amino acid derivatives (4-hydroxyphenylpyruvate and N-methylhistidine) and two unidentified metabolites (a doublet peak at 3.33 ppm and a singlet peak at 8.20 ppm) displayed significant differences. These findings suggest that alterations in energy metabolism preceding entry into high altitude may signal an individual’s susceptibility to AMS in such regions. If validated in larger cohorts, these markers would provide a basis for developing non-invasive screening methods to assess AMS susceptibility before high-altitude sojourns.

Moreover, researchers at the General Hospital of the People’s Liberation Army, led by Wang et al. (2018), explored the changes in inflammatory cytokines and acute-phase proteins under high-altitude conditions, aiming to contribute to early diagnosis of Acute Mountain Sickness. The study found significant differences in acute-phase proteins and pro-inflammatory cytokines IL-1β, IL-6, and TNF-α between the AMS and non-AMS groups. Furthermore, certain combinations of these inflammatory cytokines or acute-phase proteins could enhance the specificity of AMS diagnosis, potentially providing objective biomarker indicators for screening AMS-susceptible individuals during rapid ascent phases.

Ascending to high-altitude regions triggers a series of physiological and molecular changes, making adaptation difficult for some lowland residents. Predicting an individual’s susceptibility to Acute Mountain Sickness (AMS) prior to ascent remains challenging. A team led by Hao-Ran Guo from the Medical College of PLA (Guo et al., 2023) found that AMS-susceptible individuals and tolerant ones exhibit significant differences in inflammatory responses and immune-related biological processes at both low and moderate-high altitudes, suggesting that SAP, AAT, LTF, and HSP90-α could serve as potential biomarkers for predicting AMS in low-altitude populations.

Yu et al. (2016) from Third Military Medical University focused on objective diagnostic indicators and potential genetic polymorphisms related to AMS, particularly those involving the EPAS1 gene. Their research revealed that heart rate (≥82 beats/min), FVC (≤4.2 Lt), and Vm-BA (≥43 cm/s) level changes can serve as predictive factors for AMS accompanied by High Altitude Syndrome. Specifically, the A allele at rs4953348 locus acts as a protective factor against AMS by influencing HR and Vm-BA, while the G allele may predispose individuals to AMS with concurrent hypoxic pulmonary hypertension.

Boos et al. (2016) explored the relationship between high altitude environments, endothelial activation, inflammation, and AMS severity. They found that exposure to high altitude leads to endothelial cell activation and pro-inflammatory effects, with elevated ET-1 and IL-6 levels significantly influenced by exercise and degree of hypoxia; ET-1 was confirmed as an independent predictor of AMS and its severity.

The N Nourkami-Tutdibi team (Nourkami-Tutdibi et al., 2023) investigated changes in cytokine concentrations in blood samples and their possible association with AMS development. They discovered that VEGF levels significantly increased shortly after high-altitude exposure in AMS patients, suggesting it might be a crucial biomarker for AMS.

At the Comprehensive Rheumatology Center of Western Theater Command General Hospital’s Department of Traditional Chinese Medicine, Yang et al. (2023) constructed a Severe Acute Mountain Sickness (SAMS) prediction model using a support vector machine recursive feature elimination algorithm. The study used peripheral blood mononuclear cells from 21 volunteers exposed to very low-pressure hypoxia (VLH) conditions (406 mmHg pressure, 5,260 m elevation) and found that VLH exposure activated leukocyte gene expression, causing inversion of the CD4/CD8 ratio and interacting with genetic risk factors. Through machine learning training on 2,286 potential risk genes, they established a screening model comprising 10 significantly predictive feature genes, identifying Ephb3, Dip2B, rheumatisms, GILNT13, and SLC8A2 as five potential SAMS biomarker candidate genes.

Jia Zhi-Long et al. (Yang et al., 2022) conducted a longitudinal cohort study at the General Hospital of the People’s Liberation Army focusing on proteomics and clinical biomarkers for acute mountain sickness. They systematically analyzed AMS phenotypes, multiple clinical indicators, and AMS plasma proteome profiles of 53 participants, quantifying 1,069 proteins and validating 102 proteins. Using differential analysis, precise machine learning methods, and functional association analysis, they discovered RET protein’s key role in AMS pathogenesis and prioritized ADAM15, PHGDH, TRAF2, and TRAF2 as protective, predictive, and diagnostic biomarkers through an XGBoost model (AUC > 0.9).

In addition, Ma Li-Guang’s team from the National Research Institute for Family Planning (Ma et al., 2018) studied the spatial distribution of congenital heart disease prevalence among 4–18-year-old children in the Qinghai-Tibet Plateau and its relationship with environmental risk factors. Using Getis Ord-Gi spatial statistical methods, they uncovered a significant spatial clustering pattern of CHD prevalence, with higher rates in areas such as Yushu Tibetan Autonomous Prefecture (average elevation > 4,000 m) and Mongolian Autonomous County (average elevation > 3,600 m). Bayesian spatial binary regression analysis did not reveal a direct correlation between high altitude and CHD prevalence but identified significant associations between environmental temperature, urban accessibility index, and actual *per capita* GDP with the overall, male, female, and total CHD prevalence rates.

#### 3.2.2 Clinical information based prediction models

The clinically-informed predictive Model section summarizes studies that collectively demonstrate significant advances in predicting high-altitude adaptation, susceptibility to acute mountain sickness (AMS), fatigue state recognition, and athlete response to altitude training. The following is a summary of the core results of each research:

The research team led by Professor Wang Jiucun and Academician Jin Li from Fudan University (Li et al., 2021) developed a composite phenotype analysis method for predicting the high-altitude acclimatization capability of residents from lowland areas in China. Utilizing physiological phenotypic data from 883 Han Chinese individuals at both baseline (plain stage) and chronic (acclimatized) stages, which included 33 indicators, they innovatively combined spectral decomposition clustering algorithms with partial least squares path modeling to extract 14 representative high-altitude acclimatization composite phenotypes. The K-means algorithm was employed to reveal the physiological heterogeneity among the Han population during the acclimatization process. Further comparison showed that the model based on composite phenotypes significantly outperformed single-phenotype models in predicting changes in blood oxygen saturation during the chronic period. This method, named CompositePhenotypeAnalysis (CPA), holds significant value not only for high-altitude acclimatization studies but also for complex trait research in life health fields such as large-scale complex disease phenomics.

At the Sanitary Service Science Teaching and Research Office of the Medical Training Base at the Third Military Medical University, researchers including You et al. (2015) constructed an LVQ neural network model for predicting acute mountain sickness (AMS) susceptibility. They selected 314 healthy adults and tested them with 22 indicators encompassing both physiological and psychological aspects before entering high altitude. Following high-altitude exposure, AMS diagnoses were made according to internationally recognized criteria. Leveraging the fault-tolerance advantage of the neural network model, they built an AMS susceptibility prediction model based on preselected susceptible indicator features. Experimental results indicated that this model had a sensitivity of 95.00% in predicting individuals who would not develop AMS and an average prediction accuracy of 72.22%, validating its high predictive reliability and providing an effective tool for identifying AMS-susceptible populations in high-altitude environments.

Wang (2013) from the Computer College of Qinghai Normal University conducted a study on the application of web data mining technology in predicting AMS-susceptible populations. They applied web data mining techniques to predict AMS susceptibility, integrating and cleaning dispersed data found online, storing it in a dedicated data warehouse, and proposing a simple yet efficient AMS susceptibility prediction model.

In a study by Burtscher et al. (2004) from Innsbruck University in the United Kingdom, involving 150 healthy male and female mountaineers aged 42 ± 13 years, it was discovered that after 20–30 min of exposure to normobaric or hypobaric hypoxia, SaO_2_ values obtained through pulse oximetry could effectively distinguish AMS-sensitive from non-sensitive subjects. On average, AMS-sensitive individuals had SaO_2_ values 4.9% lower than those of non-sensitive individuals. Logistic regression analysis revealed that the relationship between SaO_2_ values and altitude could be used to predict AMS sensitivity, with their established model accurately predicting AMS susceptibility in 86% of participants.

Chen et al. (2021) from the School of Resources and Civil Engineering at Northeastern University studied the physiological and psychological information of miners working in high-altitude cold regions during fatigue states, constructing a machine learning-based fatigue recognition model. Through field testing on 45 miners working at elevations between 3,500 and 4,000 m and temperatures ranging from −5°C to −15°C, using indicators such as electrocardiogram (ECG), electromyography (EMG), pulse, blood pressure, reaction time, and lung capacity, they used Pearson correlation, t-tests, and Receiver Operating Characteristic (ROC) curves to analyze the data. Finally, they employed Support Vector Machines (SVM) and Random Forest (RF) techniques to fuse information and create a factor combination model, successfully categorizing the miners’ fatigue states, with the best fatigue classification factors being ECG-FD and EMG, and the optimal feature combination being ECG-TD + ECG-FD + EMG, where SVM models demonstrated excellent recognition performance.

Han et al. (2023) from the College of Instrumentation and Engineering at Jilin University focused on the adverse consequences athletes might experience during high-altitude hypoxic training. They developed a machine learning prediction model by collecting physiological index data from 64 professional speed skaters during sea-level and hypoxic training periods, including red blood cell count and hemoglobin concentration as variables reflecting oxygen-carrying capacity. Using maximum likelihood estimation to estimate changes in physiological indicators, they constructed a mathematical model converted into a machine learning model. Compared to polynomial curve fitting models, this machine learning model showed higher accuracy in estimating physiological variables under hypoxic training conditions, maintaining errors within 5% of measured values, effectively predicting physiological index changes in athletes under hypoxic conditions. This research provides important references for clinical medicine and human fatigue recognition in high-altitude, low-temperature, and low-oxygen environments, and is significantly valuable for optimizing high-altitude hypoxic training regimens in sports.

In summary, these studies not only improve the accuracy and reliability of prediction of AMS and other plateau adaptability problems through innovative analysis methods and techniques, but also provide scientific basis and practical tools for health management, athlete training optimization and human fatigue identification in plateau environment. Future research can further integrate the advantages of these various approaches, such as combining composite phenotypic analysis with machine learning techniques, or incorporating more dimensions of data (such as genetic information, environmental factors, etc.) into the model to build more comprehensive and personalized predictive models. In addition, with the continuous advancement of big data and artificial intelligence technology, real-time monitoring and dynamic adjustment of predictive models will become possible to provide real-time health risk assessment and intervention recommendations for participants in high-altitude activities.

#### 3.2.3 AMS prediction models based on physiological monitoring

The team led by Liu Jianchao at the Medical Innovation Research Department and Hospital Management Research Institute of the General Hospital of the People’s Liberation Army (Liu et al., 2023) constructed a medical auxiliary predictive model named SARIMA (Seasonal Autoregressive Integrated Moving Average) for predicting the occurrence of Acute Mountain Sickness (AMS) in hospitalized patients. This model identified time series characteristics of AMS hospitalized patients through retrospective analysis of historical medical records from 22,663 hospitalized patients between January 2009 and December 2018 in military hospitals. Using seasonal autoregressive integrated moving average methods, the model was trained and tested. After training with data from 2009 to 2017, the Sarma (1,1,1) (1,0,1) 12 model was used to predict data from 2018, resulting in an adjusted prediction error rate of 9.24%, which is lower than the actual value. The study revealed that AMS hospitalized patients exhibit clear cyclical and seasonal patterns, and the SARIMA model possesses good fitting capability and high short-term prediction accuracy, contributing to deeper exploration of AMS disease characteristics and providing decision-making references for healthcare resource allocation for AMS hospitalized patients.

At the Beijing Air Force Specialty Medical Center, Xiao et al. (2021) established an AMS prediction model using Radial Basis Function Neural Network for individuals adapting to high altitudes. They conducted tests on 98 personnel about to ascend rapidly to high altitude for parameters such as hemoglobin, P50, body mass index, and single nucleotide polymorphism sites in EPAS1 and EGLN1 genes. Upon rapid ascent to high altitude, they diagnosed AMS according to diagnostic standards. Based on these data, researchers developed an AMS disease diagnostic prediction model using a radial basis function neural network with a learning training accuracy of 88.0% and a matching rate with diagnosis results of 88.9%. The ROC curve area under the curve was 0.917, indicating excellent diagnostic efficiency. This finding suggests that the prediction model based on the radial basis function neural network can effectively be applied for early diagnosis of AMS.

Ye Xiaowei’s research (Ye et al., 2023) points out that wearable technology devices like smartwatches can assess cardiorespiratory health status and quantify maximum oxygen consumption (VO_2_ max), which are helpful in predicting AMS. Their investigation at low and high altitudes found that although VO_2_ max-SWT had systematic bias in estimating appropriate VO_2_ max, VO_2_ max-SWT at low altitudes could serve as an effective indicator for AMS. When combined with Red Cell Distribution Width-Coefficient of Variation (RDW-CV) at low altitudes, it could better identify individuals susceptible to AMS after acute exposure to high altitudes.

Martin et al. (2015) from Innsbruck University in the United Kingdom further propose that “physical activity” and “arterial oxygen saturation under normoxia” constitute the best combination of factors for predicting AMS incidence, correctly identifying cases up to 90% with an *R* ^2^ value of 59%. They discovered that higher levels of physical activity and lower arterial oxygen saturations were negatively correlated with the likelihood of developing AMS. Consequently, they concluded that the AMS incidence prediction model built on these two parameters has extremely high value and can serve as a reliable alternative to existing models. Notably, physical activity and arterial blood oxygen saturation are parameters easily obtainable without simulating high-altitude environments. Given this, future research should delve deeper into the impact of physical activity at varying intensities in high-altitude settings on AMS and its role in AMS prediction.

Lacey et al. (2018) have ventured to investigate the feasibility of generating a human “breathprint” using an electronic nose in high-altitude environments. This study robustly validated the applicability of electronic olfaction for volatile organic compound analysis in breath at high altitudes, thereby furnishing proof-of-concept data that substantiate the device’s potential as an objective tool for Acute Mountain Sickness (AMS) prediction and diagnosis. The technology holds considerable pragmatic value in high-altitude medicine and other contexts where hypoxia intolerance may be critical, such as in critically ill patients.

End-tidal carbon dioxide (ETCO_2_), a precise and non-invasive measure of ventilation, has been scrutinized by Thundiyil et al. (2023) with regard to its predictive power over AMS development. Their findings revealed that baseline, ETCO_2_ changes outperformed elevations in forecasting AMS symptomatology, demonstrating area under the curve (AUC) values of 0.90 (95% CI: 0.81–0.99) *versus* 0.64 (95% CI: 0.45–0.83). Notably, when, ETCO_2_ readings were ≤22 mmHg, sensitivity for predicting AMS was 100%, while specificity was 60%. Conclusively, ETCO_2_ demonstrated a significant positive correlation with altitude and a moderate positive correlation with AMS incidence, and was found to be a superior predictor of AMS compared to mere altitude measurements alone.

The intricate pathophysiological mechanisms underpinning Acute Mountain Sickness (AMS) remain enigmatic, potentially implicating adaptive or maladaptive responses from the respiratory, renal, and cerebrovascular systems to hypoxia (Barclay et al., 2021). Barclay et al. thus delved into the predictive roles of respiratory alkalosis and posterior cerebral artery dilation on AMS severity during a 10-h normobaric hypoxia exposure. Their findings revealed that compared to normoxic conditions, blood flow velocity (LSL) in the internal carotid artery (ICA) and vertebral artery (VA), ventilation rates, venous and urine pH levels, blood flow, and vessel diameters all increased under hypoxia, while venous bicarbonate and creatinine concentrations significantly decreased (*p* < 0.001). They further observed significant correlations between AMS severity and parameters such as blood pH, sodium concentration, changes in VA diameter, and fluid balance (*p* < 0.05). Through stepwise regression analysis, they identified blood pH (β-coefficient = 0.589, *p* < 0.001) and enlarged VA diameter (β-coefficient = 0.418, *p* = 0.008) as substantial predictors of AMS severity within their study population, accounting for 62% of the peak LLS variance [F (2,20) = 16.1, *R* ^2^ = 0.617, *p* < 0.001; *n* = 24]. This suggests that the extent of respiratory alkalosis and cerebrovascular dilatation may be critical factors in the early stages of AMS onset.

Hamlin and Ainslie (2010), building upon these insights, scrutinized whether physiological variables measured at sea level could forecast AMS at actual high altitudes, using the Lake Louise Score as a diagnostic tool. Employing multiple linear regression, they found mean sea-level blood pressure to be the sole statistically significant predictor of altitude-induced AMS (*p* < 0.01), explaining 45% of the variability in AMS incidence at high elevations. Although this finding underscores the predictive utility of mean sea-level blood pressure, it is apparent that additional contributing factors are involved in AMS development at high altitudes, necessitating further research to elucidate their specific roles.

In research carried out at the Life Sciences Institute within the National Defense Medical Center, Wei Zhiyuan and associates have devised a sophisticated Acute Mountain Sickness (AMS) risk assessment model (Wei et al., 2022). Leveraging machine learning algorithms to meticulously track real-time physiological and environmental data from subjects in high-altitude conditions, they have successfully demonstrated its potential for accurate AMS prediction and prevention. This groundbreaking work serves as a foundational theoretical underpinning for the development of wearable devices with integrated AMS risk functionality.

In an effort to tackle this multifactorial issue, Ding et al. (2023) constructed an acute mountain sickness risk prediction model utilizing machine learning algorithms. Their retrospective cohort study collected AMS surveillance data from workers engaged in the National Grid’s Sichuan-Tibet transmission project between 1 January 2019, and 31 December 2020. The dataset was divided into training and test sets at a 7:3 ratio and subjected to 10-fold cross-validation to enhance the model’s generalizability. Four models were compared, including the random forest (RF), with performance assessed via area under the curve (AUC) and accuracy metrics. The cohort comprised 10,956 workers, predominantly male (95.27%) with a mean age of 36.13 ± 10.49 years, and reported an overall AMS incidence rate of 15.58%. The results demonstrated that the random forest model outperformed other models in predicting AMS risk. By accurately predicting AMS risk, workers can implement preventative measures to mitigate its occurrence effectively.

At Beijing Institute of Technology’s School of Automation, Chen Jing and fellow researchers from her team have harnessed the power of medical Internet of Things (IoT) technology to conduct intelligent, dynamic risk assessments for acute mountain sickness (Chen et al., 2023) They have effectively validated an event-driven data scheduling and signal processing methodology that enables real-time AMS risk evaluation on resource-constrained wearable devices, thereby significantly advancing the prospects for personalized healthcare applications in this domain.

Concurrently, Wang Lei’s research group at the Institute of Engineering Medicine at Beijing Institute of Technology has tackled the issue of subjectivity inherent in prevailing AMS assessment methodologies (Wang et al., 2023). They introduced an innovative approach combining Long Short-Term Memory (LSTM) learning models with Slow Feature Analysis to provide a more objective evaluation of individual hypoxia tolerance and AMS vulnerability. Through meticulous monitoring and analysis of a diverse array of physiological parameters, they have successfully engineered a highly precise hypoxia tolerance assessment model. This model boasts an impressive classification accuracy of 85.71% and achieves an area under the Receiver Operating Characteristic (ROC) curve of 0.925, showcasing superior performance across multiple evaluation benchmarks.

In summary, the research into predictive models for high-altitude illnesses is not only pivotal for enabling early warnings and precise management but also instrumental in optimizing resource allocation, enhancing our understanding of the underlying mechanisms of high-altitude diseases, and furnishing robust scientific backing for clinical decision-making. Ultimately, these advancements contribute significantly to reducing the incidence of high-altitude illnesses and associated complications, ensuring the safety and health of individuals operating in challenging high-altitude environments.

### 3.3 TCM’s contribution to the study of predicting high altitude illnesses

Traditional Chinese Medicine (TCM), a treasure trove of China’s traditional medical practice, embodies the profound historical accumulation and wisdom of Chinese civilization. Its preventive medical philosophy dates back to antiquity, as evidenced by the principle of “treating disease before it arises” in the “Huangdi Neijing”, which underscores the importance of maintaining health through adjustments and preventative measures before illness occurs. In the “Plain Questions: The Great Treatise on Regulating the Four Seasons and Nurturing Life” within this classic text, an explicit emphasis is placed on prioritizing prevention in healthcare maintenance (Li et al., 2023).

Grounded in TCM’s “treating disease before it arises” theory, combined with etiology-pathogenesis theories and constitutional differentiation systems, TCM scholars employ the comprehensive diagnostic approach of observing, listening, questioning, and palpating (the “Four Examinations”) to identify AMS (Acute Mountain Sickness) susceptible population characteristics. By examining and analyzing the distribution and evolution patterns of syndromes, they have unearthed unique, TCM-characteristic AMS risk prediction factors. These findings have culminated in the development of a distinct TCM-based AMS prediction model, aiming to address the limitations of current AMS prediction methods, thereby enhancing accuracy and efficiency (Li et al., 2023; Wang et al., 2018).

In-depth research has been conducted across four aspects of TCM diagnosis for this distinctive predictive system:

(1) Observation (Wang Zhen): TCM posits that Qi deficiency and Yang deficiency are fundamental syndrome types underlying AMS, characterized mainly by Qi deficiency and blood stasis. Through meticulous observation of patients’ demeanor, physique, and tongue manifestations, psychological states such as chronic depression, anxiety, or excessive mental strain may exacerbate AMS progression (Boos et al., 2018; Zhang et al., 2019). Additionally, specific physical traits like a short and stout stature, neck and shoulder contours, along with changes in tongue coating color, moisture, and thickness, might indicate heightened susceptibility to AMS (Zhen and Zhou, 2004; Zhen et al., 2023).
(2) Auscultation and Olfaction (Wen Zhen): By perceiving a patient’s breath and voice, one can assess the likelihood of AMS occurrence. In high-altitude hypoxic environments, pathogenic wind easily invades the body, primarily affecting lung function, manifesting as coughing, shortness of breath, and wheezing. Adequate respiratory function is crucial for adapting to high altitudes; thus, ventilatory function tests serve as effective predictors of high-altitude illnesses (Ledochowski and Fuchs, 2003). Moreover, individuals with chronic obstructive pulmonary disease or upper respiratory infections are less suited for entering high-altitude regions (Wu et al., 2014).
(3) Inquiry (Wen Zhen): Systematically inquire about a patient’s family history, past medical history, high-altitude residence history, and lifestyle factors to pinpoint AMS susceptibility elements. For instance, poor dietary habits leading to obesity have been identified as independent risk factors for acute high-altitude cerebral edema and high-altitude heart disease (Wang et al., 2006).
(4) Palpation (Qie Zhen): Through pulse diagnosis, evaluate an individual’s visceral functions and the state of Qi and blood. Some studies have attempted to analyze features such as pulse intensity, rhythm, and rate in a high-altitude environment to predict the risk of high-altitude illness. A weak, slow, or irregular pulse might reflect insufficient Qi and blood, dysfunctional viscera, and suggest increased vulnerability to acute mountain sickness (Wang et al., 2023).
(5) Herbal Prevention and Treatment: TCM has a long history of application in preventing and treating acute mountain sickness. Multiple studies have explored the mechanisms by which herbal medicines modulate AMS susceptibility, reducing its incidence and progression by regulating Qi and blood circulation and strengthening organ functions. Certain herbal formulations, including Tibetan compound formulas, Rhodiola rosea, Cordyceps sinensis, its mycelium, and Potentilla fruticosa, have demonstrated promising therapeutic effects due to their anti-hypoxia, anti-inflammatory, and oxygen transport-enhancing properties, potentially improving individuals’ altitude adaptability (Jia and Yang, 1999; Li et al., 2015; Deng et al., 2016). Indigenous herbs from high-altitude regions present extensive research prospects and practical value in the prevention and treatment of both acute and chronic high-altitude illnesses.

In summary, TCM plays a significant role in the research of predicting acute mountain sickness, particularly in risk assessment, personalized management, and adjunctive therapy. However, further scientific validation and refinement are needed to establish its efficacy and reliability in AMS prediction.

## 4 Problems and challenges in the prediction of AMS

In the realm of high-altitude illness prediction research, a series of critical challenges remain that are vital for enhancing the precision and reliability of predictions. The following issues stand out as central to this field.

### 4.1 Interplay of multiple factors

The occurrence of high-altitude illnesses is governed by a multifaceted interplay of factors encompassing individual physiological characteristics, environmental exposures across various dimensions (Huang, 2022; Ngiam et al., 2023). While constructing an effective predictive model necessitates comprehensive consideration and integration of these variables, accurately quantifying the weight relationships and interaction patterns among them remains a daunting task.

### 4.2 Data quality and scale constraints

The development of reliable prediction models relies heavily on a large volume of high-quality data, including individuals’ baseline biostatistical information and real-time physiological parameters. However, acquiring and integrating such data in practical settings, especially under challenging high-altitude conditions, proves difficult, with ensuring data completeness and accuracy being a significant hurdle.

### 4.3 Lack of standardized indicators

Currently, there is no consensus on the indicators and standards employed for predicting high-altitude illnesses; the divergent choices made in different studies impede effective comparison of results (Macholz et al., 2018). Thus, establishing a standardized set of indicators that serve as the foundation for developing and evaluating high-altitude illness prediction models is imperative.

### 4.4 Limitations in model generalizability

Ideal prediction models should exhibit robust generalization capabilities across diverse environments and populations. Nevertheless, due to geographical variations, racial genetic backgrounds, and other factors, the adaptability of such models may be constrained when applied to different regions and populations (Wei et al., 2022).

### 4.5 Insufficient interpretability

While some prediction models demonstrate high predictive accuracy, they often lack detailed explanations regarding the underlying pathophysiological mechanisms driving specific individual predictions. This dearth of interpretability can potentially constrain clinical decision-making processes and the implementation of targeted intervention strategies.

### 4.6 Absence of dynamic prediction windows

Given that the onset of high-altitude illnesses unfolds as a continuous process, whereas current prediction models predominantly rely on static data, capturing dynamic changes and translating them into real-time precise predictions remains a key challenge yet to be overcome.

Confronted with these challenges, future research endeavors should strive to optimize data collection methods and quality control measures, establish a unified framework of predictive indicators and standards, enhance model generalizability, and develop more flexible and dynamically adaptive prediction models to better serve the needs of early warning systems for high-altitude illnesses. Moreover, interdisciplinary collaboration and shared data resources will play a pivotal role in catalyzing breakthrough advancements in high-altitude illness prediction research.

## 5 Prospects for predicting AMS with AI, big data, and IoT

The rapid development of emerging technologies such as artificial intelligence (AI), big data, and the Internet of Things (IoT) is ushering in unprecedented opportunities and breakthroughs in the field of high-altitude illness prediction. These advanced technologies hold promise to significantly enhance the accuracy of prediction models for high-altitude illnesses, improve individualized management effectiveness, and bolster real-time monitoring capabilities, thereby effectively reducing the incidence of these illnesses and enhancing treatment outcomes (Wang et al., 2023; Ye et al., 2023; Karinen et al., 2010). However, their application in predicting AMS has been considered unreliable in the past due to multiple factors. Therefore, the use of AI, ML, DL and other methods requires improved prediction accuracy and reliability, while a large data pool is required to apply these modern technologies to develop a prediction model with high sensitivity and specificity.

### 5.1 Revolutionizing data collection and processing

IoT technology enables continuous and real-time monitoring of physiological indicators and environmental parameters for individuals in high-altitude settings. Through a variety of sensors and wearable devices, more comprehensive and accurate data can be gathered, providing an authentic reflection of an individual’s health status in high-altitude environments.

### 5.2 Deep mining and analysis of big data

Leveraging big data analytics tools and techniques, the exploration and in-depth analysis of vast amounts of individual data can help identify key characteristics and biomarkers closely related to the pathogenesis and progression of high-altitude illnesses. This will powerfully drive the creation of more precise and robust prediction models for these conditions.

### 5.3 Personalized prediction and precision management

The application of AI technology allows for the establishment of personalized high-altitude illness prediction models based on individual characteristics and historical data. Machine learning and deep learning algorithms can dynamically assess an individual’s disease risk and devise targeted prevention and intervention strategies.

### 5.4 Real-time monitoring and early warning system construction

Integrating IoT technology with advanced AI algorithms, a real-time monitoring and early warning system for high-altitude illnesses can be established. By instantaneously analyzing real-time individual data, potential health risks can be promptly detected and signals issued, ensuring swift response measures are taken.

### 5.5 Decision support systems

AI applications also facilitate the development of efficient decision support tools and platforms that provide precision treatment recommendations and intervention guidance based on data analysis for clinicians and healthcare professionals, further strengthening the scientificity and efficacy of clinical decision-making.

### 5.6 Interdisciplinary collaboration and resource sharing

The promotion and application of new technologies are inseparable from close interdisciplinary collaboration and resource sharing. Joint efforts by healthcare institutions, research organizations, and technology enterprises, contributing their specialized knowledge and data resources, will greatly propel progress in high-altitude illness prediction research and practical applications.

In summary, innovative technologies like AI, big data, and IoT present broad application prospects and immense potential in high-altitude illness prediction. Future research priorities will focus on more efficient data collection and analysis, deeper personalization of predictions and management, refining real-time monitoring and early warning systems, and reinforcing interdisciplinary cooperation and resource-sharing mechanisms. Ultimately, these advancements aim to offer more precise and effective preventive and therapeutic methods for populations in high-altitude environments, ensuring their health and safety.

## 6 Conclusion

With the growing human exploration and increased activities in high-altitude environments, research into predicting Acute Mountain Sickness (AMS) has become increasingly pivotal. This review systematically synthesizes key advancements in AMS prediction studies over recent years and thoroughly examines the application value and challenges of novel technologies within this domain. Initially, we delve deeply into the pathophysiological mechanisms underlying AMS, elaborating extensively on core aspects such as adaptive responses to hypoxia, hemodynamic alterations, increased blood viscosity, and the resultant spectrum of tissue damage and complications. These foundational theories provide a robust theoretical underpinning for constructing scientifically sound and rational AMS prediction models. Subsequently, we analyze contemporary cohort research targeting AMS, encompassing the characteristics of diverse study populations, the variety in research scopes and methodologies, and the critical contribution these empirical findings make in elucidating key factors contributing to AMS onset. These datasets furnish substantial and robust data support for constructing and refining predictive models. Next, we meticulously outline the development process of AMS prediction models, which includes the significance of feature selection, strategies for model construction, and methodological considerations for model validation and performance assessment. Simultaneously, we underscore the crucial role that prediction models play in enabling early warning of AMS, personalized risk assessments, judicious allocation of medical resources, and informed clinical decision-making. Finally, we explore the promising prospects of emergent technologies like artificial intelligence, big data analytics, and the Internet of Things (IoT) within the realm of AMS prediction. We also highlight potential issues and challenges encountered in practical applications, including the determination of multi-factorial weightings, requirements for data quality and quantity, establishment of unified predictive indicators and standards, enhancement of model generalizability, and interpretability of prediction outcomes. Such cutting-edge technologies are poised to significantly enhance the precision and real-time responsiveness of AMS prediction models, thereby driving advancements in preventative and therapeutic strategies, ultimately better safeguarding the life safety and health standards of individuals operating in high-altitude settings.

In summary, this review provides a comprehensive overview of the latest trends and technological developments in acute mountain sickness prediction research while explicitly identifying key areas for future attention and resolution. It aims to further propel the progression of AMS prediction studies, offering more precise and efficacious scientific and technological support for individual health management in high-altitude environments.
